# Cadmium Highlights Common and Specific Responses of Two Freshwater Sentinel Species, *Dreissena polymorpha* and *Dreissena rostriformis bugensis*

**DOI:** 10.3390/proteomes12020010

**Published:** 2024-03-26

**Authors:** Florence Bultelle, Aimie Le Saux, Elise David, Arnaud Tanguy, Simon Devin, Stéphanie Olivier, Agnès Poret, Philippe Chan, Fanny Louis, Laurence Delahaut, Sandrine Pain-Devin, Romain Péden, David Vaudry, Frank Le Foll, Béatrice Rocher

**Affiliations:** 1 UMR-I 02 INERIS-SEBIO, UFR ST, Scale FR-CNRS 3730, Le Havre Normandie University, 76063 Le Havre, Francebeatrice.rocher@univ-lehavre.fr (B.R.); 2 UMR-I 02 INERIS-SEBIO, UFR SEN, Reims Champagne-Ardenne University, 51100 Reims, France; elise.david@univ-reims.fr (E.D.);; 3 UMR 7144, CNRS, Station Biologique de Roscoff, Sorbonne University, 29680 Roscoff, France; atanguy@sb-roscoff.fr; 4 LIEC, CNRS, UFR SCIFA, Lorraine University, 57000 Metz, France; simon.devin@univ-lorraine.fr (S.D.);; 5 INSERM US 51, CNRS UAR 2026, HeRacLeS, Rouen Normandie University, 76821 Mont-Saint-Aignan, France; 6 PISSARO IRIB, Rouen Normandie University, 76821 Mont-Saint-Aignan, France; 7 INSERM U982 DC2N, Rouen Normandie University, 76821 Mont-Saint-Aignan, France

**Keywords:** quagga mussel, zebra mussel, proteoforms, stress, biomonitoring

## Abstract

Zebra mussel (ZM), *Dreissena polymorpha*, commonly used as a sentinel species in freshwater biomonitoring, is now in competition for habitat with quagga mussel (QM), *Dreissena rostriformis bugensis.* This raises the question of the quagga mussel’s use in environmental survey. To better characterise QM response to stress compared with ZM, both species were exposed to cadmium (100 µg·L^−1^), a classic pollutant, for 7 days under controlled conditions. The gill proteomes were analysed using two-dimensional electrophoresis coupled with mass spectrometry. For ZM, 81 out of 88 proteoforms of variable abundance were identified using mass spectrometry, and for QM, 105 out of 134. Interestingly, the proteomic response amplitude varied drastically, with 5.6% of proteoforms of variable abundance (DAPs) in ZM versus 9.4% in QM. QM also exhibited greater cadmium accumulation. Only 12 common DAPs were observed. Several short proteoforms were detected, suggesting proteolysis. Functional analysis is consistent with the pleiotropic effects of the toxic metal ion cadmium, with alterations in sulphur and glutathione metabolisms, cellular calcium signalling, cytoskeletal dynamics, energy production, chaperone activation, and membrane events with numerous proteins involved in trafficking and endocytosis/exocytosis processes. Beyond common responses, the sister species display distinct reactions, with cellular response to stress being the main category involved in ZM as opposed to calcium and cytoskeleton alterations in QM. Moreover, QM exhibited greater evidence of proteolysis and cell death. Overall, these results suggest that QM has a weaker stress response capacity than ZM.

## 1. Introduction

Aquatic environments are subject to extensive perturbations caused by abiotic factors such as temperature or oxygen levels and pollution. Assessing ecosystem health is crucial to understanding the impact of human activity and enhancing the preservation and remediation of natural habitats. In the European Union, this necessity, first underlined by the scientific community, has been reinforced by legally binding directives aimed at improving water quality. The Water Framework Directive (2013/39/EC) introduced new environmental quality standards for priority substances and certain other pollutants. It also set 2027 as the target date for achieving good status for water bodies. Currently, the definition of “good status” is based on chemical and ecological data but does not include an assessment of the health of biota via ecotoxicological data. A European Consortium was established to enhance regulations by utilising innovative biological monitoring techniques. The consortium recommends the development of integrated effect-based methods for environmental survey. To this end, it is crucial to identify the mechanisms and molecular players involved in the stress response of the species under consideration.

Sentinel or bioindicator species are chosen to represent the biota in biomonitoring studies. The species selected must meet certain biological and ecological criteria, such as being representative of its environment and of the collection site, having the capacity to bioaccumulate pollutants over time and having a wide distribution area. Technical criteria are also considered, i.e., ease of identification and year-round availability for collection. In coastal and marine environments, *Mytilus* species are the most widely used. They are sedentary filter-feeders and have a good capacity to bioaccumulate pollutants, combined with a low capacity for biotransformation. Zebra mussel (ZM), *Dreissena polymorpha* (Pallas, 1771) is a very similar biological model that closely resembles *Mytilus* and has been used as a counterpart in freshwater ecosystems since the 1970s [1]. ZM are sedentary filter-feeding organisms with high filtration activity; they bioaccumulate many contaminants. They originate from the Ponto–Caspian area and have largely colonised Western Europe and also North America, especially the Great Lakes region [2]. More recently, the quagga mussel (QM), *Dreissena rostriformis bugensis* (Andrusov, 1897), a closely related dreissenid also native to the Ponto–Caspian region, has in turn become invasive in Western Europe and North America [3]. The coexistence of the two species within the same area and the QM’s displacement of ZM in some locations raises the question of using the QM as a counterpart sentinel species.

In 2022, Karatayev and Burlakova reviewed the existing knowledge on these two dreissenids [4]. In brief, ZM and QM exhibit physiological differences including variances in temperature and salinity tolerances, byssal thread attachment, growth rate, respiration rate, assimilation efficiency, and reproductive outcomes. Although QM has been found to be less tolerant of high temperatures than ZM, it withstands low temperatures better. Additionally, QM features a higher filtration rate but a lower respiration rate relative to ZM. Differential xenobiotic accumulation has been observed in both species in biomonitoring studies. For instance, Schäfer and collaborators reported that QM accumulated more organochlorine pesticides, experienced greater DNA damage, and exhibited lower levels of HSP stress proteins compared to ZM [5]. In contrast, Kerambrun and collaborators recorded a higher bioaccumulation of metals (Cd, Cu, Ni, Pb and Zn) and a higher gene expression level of the antioxidant enzyme catalase in ZM than in QM during a field study at a site shared by both species [6]. In a comparison of haemocyte oxidative activity, QM was shown to have a higher level of ROS than ZM [7]. Differences in biomarker responses were also noted between the two species after controlled metal exposure [8,9]. Energy allocation and antioxidant capacity varied between QM and ZM, wherein QM displayed more cellular damage upon cadmium exposure [8]. However, Farkas et al. (2017) observed no interspecies variation when comparing the responses of different biomarkers (such as EROD activity, lipid peroxidation, DNA damage, metallothionein, and vitellogenin) in ZM and QM populations from contaminated sites in Hungary [10]. Therefore, the above-mentioned disparities impede the mere transfer of biomarkers developed in ZM to QM and emphasise the necessity of further characterising contaminant effects on QM.

In this study, in order to compare the biological responses of the two species, we exposed QM and ZM to a single model toxicant under identical, controlled conditions. Cadmium was selected due to its well-known effects and high toxicity. Furthermore, cadmium is one of the four priority metals of the Water Framework Directive and is widely distributed in the aquatic environment [11]. Environmental cadmium concentrations in water can occasionally reach very high levels, such as 40 µg·L^−1^ in the Longjiang and Liujiang rivers in China [12] and 100 µg·L^−1^ in the water of the upper Malwathu Oya in Sri Lanka [13]. Here, proteomic comparisons were conducted on gills, a tissue involved in respiration, feeding, ion regulation, and excretion of metabolic products. Cadmium can be absorbed through the digestive tract in aquatic organisms, but part of its internalisation also occurs in permeable areas like gills, where cadmium borrows from metal or calcium membrane transporters.

The aim of this study was to improve the understanding of (i) *Dreissena* species and (ii) the sensitivity and responses of *Dreissena polymorpha* and *Dreissena rostriformis bugensis* in a biomonitoring perspective. By exploring the proteomic level, the mechanistic basis of the different ecophysiological profiles can be evidenced. Mussels from both species were exposed to cadmium at a high but realistic concentration of 100 µg·L^−1^ for 7 days. Two-dimensional electrophoresis was performed to investigate the differential expression profile of gill proteins. Variant proteoforms were identified by means of mass spectrometry using species-specific transcriptomic data, and then classified into functional categories. Some of these functional categories are common to both species, pointing to a shared response to heavy metal, whereas others are unique to each species, offering new insight into the species-specific aspects of the response to cadmium.

## 2. Materials and Methods

### 2.1. Organism Collection

Mussels were hand collected in February 2017. ZM were collected in the Meuse channel (Euville, France, N 48°44′03″, E 05°39′45″) and QM in the Saône river (Seurre, France, N 47°00′03″, E 05°08′34″). Individuals of both species were retrieved by section of the byssal thread. Mussels were quickly brought to the laboratory in aerated field water and mussel shells were gently cleaned to remove any organic deposits. Water physico-chemical parameters were measured onsite at the time of sampling. Cadmium concentrations were measured in samples of water with flameless atomic absorption spectrometry using SpectrAA 220 Zeeman (Varian, Mulgrav, The Netherlands) and revealed no cadmium contamination at both sites [14].

### 2.2. Acclimation

Mussels were grouped per species in tanks containing aerated field water at field temperature. During the first three days, water was gradually replaced by spring water (Cristaline^®^, Aurèle spring, Alma, Jandun, France). Acclimation continued for 21 days in aerated water maintained at field temperature. An artificial photoperiod was applied based on natural conditions. During this period, water was totally renewed twice a week, and mussels were fed with a fresh diet of Chlorella and Senedesmus (Greensea) at the rate of 106 cells/individual/day. Water physico-chemical parameters, checked daily, were stable (temperature 4.8 °C; pH 8.2).

### 2.3. Exposure Design and Tissue Sampling

After 21 days of acclimation, for each species, mussels from the different tanks were pooled and separated again in order to define 2 experimental conditions: 7 days of cadmium exposure and control. A stock solution of CdCl_2_ at 1 mg·L^−1^ acidified with 0.3% nitric acid was used and diluted to a final concentration of 100 µg·L^−1^ in media. Meanwhile, control mussels were kept in spring water (Cristaline^®^, Aurèle spring). The animals were maintained in aerated water at field temperature, and subjected to an artificial photoperiod consistent with the acclimation condition. Every day, water was renewed (control and exposed media) and mussels were fed according to the rate used during the acclimation phase. Water parameters were monitored daily. During the experiment, temperature, pH, and conductivity did not change between groups and no mortality was observed (Figure S1).

Water Cd concentrations were measured twice a day, immediately prior to the renewal and 2 h later. Actual water Cd concentrations reached 52.8 ± 2.1 µg·L^−1^ 2 h after the renewal and decreased to 14.7 ± 3.1 µg·L^−1^ within the 24 h for ZM and 41.4 ± 3.8 µg·L^−1^ to 6.9 ± 0.1 µg·L^−1^ for QM. After 7 days, cadmium accumulation was measured in mussels (*n* = 12). Soft tissues were dried at 60 °C for 24 h and digested in nitric acid 65% for 48 h at 80 °C. Metal quantifications were performed in the resulting acid solutions (adjusted to 3 mL with deionised water) using flameless atomic absorption spectroscopy, as described in Amiard et al. [14]. Results are expressed in µg·g^−1^ of dry weight. For 2DE analysis, the gills of 12 mussels were sampled for each group: control zebra mussels (ZM-control), exposed zebra mussels (ZM-Cd), control quagga mussels (QM-control), and exposed quagga mussels (QM-Cd). The tissues were dissected, flash-frozen in liquid nitrogen and stored at −80 °C until analysis. Mussels were measured and weighted at the time of the sampling and no difference in the condition index was observed between exposed and treated groups for both species (ZM-Cd = 0.241 ± 0.011, ZM-control = 0.242 ± 0.009; QM-Cd = 0.225 ± 0.009; QM-Control = 0.227 ± 0.007).

### 2.4. Two-Dimensional Gel Electrophoresis and Mass Spectrometry Identification

Protein extraction was performed as described by Péden et al. [15]. Briefly, gills (*n* = 12) were homogenised with a lysis buffer containing 7 M urea, 2M thiourea, 2% CHAPS, 32 mM dithioerythritol, 0.02% pharmalyte 3–10 NL, antiproteases (PierceTM Protease Inhibitor), 50 mM TRIS, pH 6.8. and, for IEF (isoelectrofocusing), a total of 600 µg of proteins per sample were loaded on non-linear wide-range immobilised pH gradient strips (IPG strip pH 3–10, NL/18 cm, GE Healthcare). After IEF and equilibration steps, SDS-PAGE electrophoresis were conducted with two Protean plus Dodeca-Cell (Bio-Rad, Hercule, CA, USA) allowing a parallel run of 24 large gels (12% polyacrylamide, 20 cm × 20 cm × 1.5 cm).

After colloidal blue staining and scanning, gels exhibiting migration impairment were excluded and gel images were analysed using Delta 2D (Decodon, Gmbh). For each spot, normality and homoscedasticity were checked (Shapiro test and F-test). Normality was always observed, but not homoscedasticity. Consequently, normalised volumes were compared between the control group and the group of mussels exposed to cadmium for each spot and each species, using either a Student *t*-test or a Welch *t*-test. Spots with a *p*-value below 0.05 were considered as differentially abundant expressed and selected for further analysis using mass spectrometry. No risk correction was applied to the computed *p*-values since spots are considered independent from each other.

### 2.5. Mass Spectrometry and Protein Identification

Mass spectrometry and protein identification were performed on spots of interest after trypsin digestion (Promega, Charbonnières-les-Bains, France) as described [15]. Peptides were analysed with a nano-LC1200 system coupled to a 6545XT AdvanceBio Q-TOF mass spectrometer (Agilent Technologies, Santa Clara, CA, USA). Briefly, peptides were enriched and desalted on a 160 nL C18 reversed-phase trap column and separated on a Zorbax C18 column (75 µm inner diameter × 150 mm long, 5 µm particle size and 30 nm pore size; Agilent Technologies). MS/MS peak lists were extracted and analysed with the Xtandem pipeline (http://pappso.inra.fr/bioinfo/xtandempipeline/, accessed on 10 August 2023) using transcriptomic data from *Dreissena polymorpha* and *Dreissena rostriformis bugensis* [16]. Proteins were further identified by searching in the non-redundant protein sequence database with the Blastp (Basic Local Alignment Search Tool) of NCBI (https://blast.ncbi.nlm.nih.gov/Blast.cgi/, accessed on 10 August 2023).

### 2.6. Functional Protein Analysis

Functional study of proteins was performed according to UniProt (https://www.uniprot.org/, accessed on 10 August 2023), Genecards (https://www.genecards.org/, accessed on 10 August 2023), GeneOntology (https://amigo.geneontology.org/, accessed on 10 August 2023), and KEGG (Kyoto Encyclopedia of Genes and Genomes) (https://www.genome.jp/kegg/, accessed on 10 August 2023) database analyses. Association network analysis of proteins was conducted using STRING (Search Tool for the Retrieval of Interacting Gens/Proteins, https://string-db.org, accessed on 6 September 2023 [17]). For more details, the interaction map was generated from the web-based search STRING v11.0 database using default settings.

## 3. Results

### 3.1. General Overview

**Figure 1:**
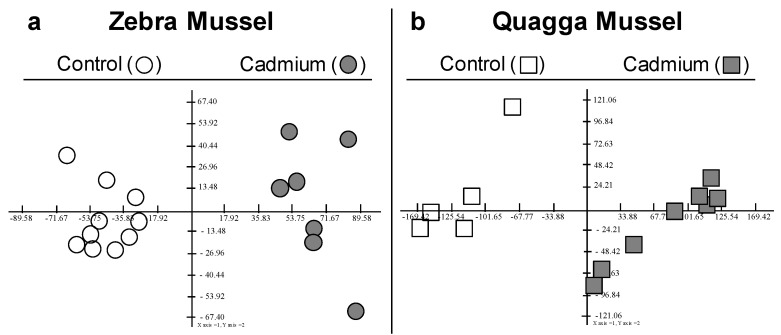
F1F2 Factorial planes of Principal Component Analysis (PCA) computed on normalised spot volume. (**a**): PCA on the 88 spots × 17 gels obtained for ZM. F1 = 34.5%, F2 = 10%. (**b**): PCA on the 134 spots × 13 gels obtained for QM. F1 = 49.2%, F2 = 9%.

In order to better understand the responses and sensitivities of *Dreissena polymorpha* (ZM) and *Dreissena rostriformis bugensis* (QM) as sentinel organisms, exposure to cadmium was performed under laboratory conditions and the gill proteomes were analysed using two-dimensional electrophoresis separation (Figures S2 and S3). The differentially abundant proteoforms (DAPs) were selected for mass spectrometry analysis, with identifications based on species-specific transcriptomic data. The study investigated the effects of a 7-day exposure to cadmium at an initial concentration of 100 µg·L^−1^ on the two species while maintaining a control group. Metal bioaccumulation was measured in both species at the end of the experiment. Significantly different concentrations were observed between the two species, with QM showing 37.2 ± 9.6 µg·g^−1^ dry weight and ZM showing 14.9 ± 3.8 µg·g^−1^ dry weight. This outcome is in line with a prior study that reported a higher uptake rate in QM than in ZM [18]. Principal component analysis (PCA) performed on the proteomic dataset clearly discriminated cadmium-exposed and control mussels in both species (Figure 1).

**Figure 2:**
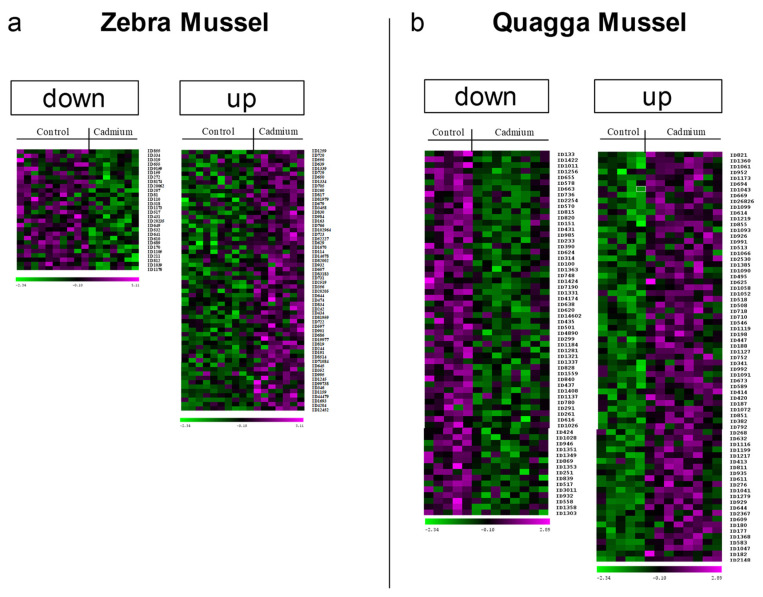
Heat maps of the differentially abundant gill proteoforms after cadmium exposure for (**a**) zebra and (**b**) quagga mussel. After a 7-day exposure to cadmium (100 µg·L^−1^), gill proteins from exposed and control mussels were extracted and separated by 2DE. Images were analysed using Delta 2D (Decodon, Gmbh). The heat maps show the 60 up-regulated and 28 down-regulated spots in zebra mussels, and the 71 up- and 63 down-regulated spots in quagga mussels.

For the detailed results of the ZM gill proteomics, the gel images were merged into a master gel displaying 1561 proteoforms. Of these, 88 exhibited a significant difference in protein abundance between exposed and control animals. In total, 60 were up-regulated and 28 were down-regulated (Figure 2a). Mass spectrometry allowed for the identification of 81 DAPs (Table S1). For QM, 134 of the 1419 proteoforms detected exhibited a significant difference in protein abundance between exposed and control animals, with 71 up-regulated and 63 down-regulated (Figure 2b). In total, 105 DAPs were identified by mass spectrometry (Table S2).

### 3.2. DAPs Classification and GO Term Enrichment Analysis

Relationships between DAPs were further investigated by using protein databases, including STRING database version 11.0 (https://string-db.org/, accessed on 6 September 2023), GeneCards (https://www.genecards.org/, accessed on 10 August 2023), UniProtKB/Swiss-Prot (https://www.uniprot.org/, accessed on 10 August 2023), GeneOntology (https://amigo.geneontology.org/, accessed on 10 August 2023) and KEGG (Kyoto Encyclopedia of Genes and Genomes) (https://www.genome.jp/kegg/, accessed on 10 August 2023). Determining a sole protein classification can be challenging due to their involvement in multiple cellular metabolic processes. Additionally, many proteins are multitasking or of limited knowledge. Based on our data, we classified the DAPs into five functional categories: chaperone, processing, folding, and degradation (Table 1A); calcium and cytoskeleton alterations (Table 1B); redox and detoxification (Table 1C); energy and metabolism (Table 1D); transcription and translation (Table 1E).

**Figure 3:**
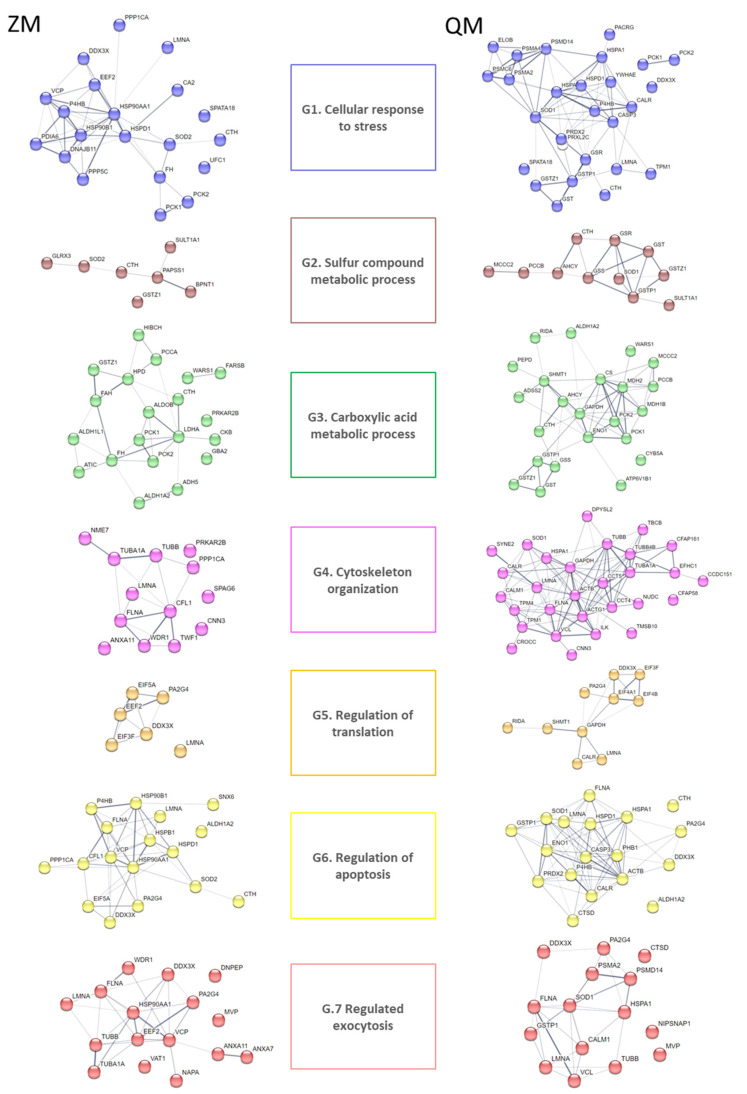
STRING analysis of cadmium-induced DAPs for zebra (ZM) and quagga (QM) mussels. Protein–protein interaction networks were constructed using the STRING database (https://string-db.org, accessed on 6 September 2023) [17]). Nodes in the network represent DAPs. Protein abbreviations are reported in Table 1. Confidence in protein interactions is shown by the thickness of the lines connecting each node. G refers to Gene Ontology terms: G1. GO:0033554_Cellular response to stress; G2. GO:0006790_Sulfur compound metabolic process; G3. GO:0019752_Carboxylic acid metabolic process; G4. GO:0005856_Cytoskeleton and G5. GO:0006417_Regulation of translation; G6. GO:0042981_Regulation of apoptotic process; G7. GO:0045055_Regulated exocytosis.

Additionally, a GO term enrichment analysis was performed, allowing the association of biological processes with each DAP (Table S4, Figures S4 and S5). The analysis results highlighted seven major biological processes, as follows: G1.GO:0033554_Cellular response to stress (21 DAPs for ZM, 30 DAPs for QM), G2.GO:0006790_Sulfur compound metabolic process (7 DAPs for ZM, 12 DAPs for QM), G3.GO:0019752_Carboxylic acid metabolic process (20 DAPs for ZM, 27 DAPs for QM), G4.GO:0005856_Cytoskeleton (14 DAPs for ZM, 35 DAPs for QM), G5.GO:0006417_Regulation of translation (7 DAPs for ZM, 11 DAPs for QM), G6.GO:0042981_Regulation of apoptotic process (18 DAPs for ZM, 23 DAPs for QM) and G7.GO:0045055_Regulated exocytosis (17 DAP for ZM, 20 DAPs for QM) (Figure 3).

Cadmium is known to interfere with calcium signalling pathway. Thus, we searched for calcium-dependent proteins in the DAPs. At least 17 DAPs were found to be calcium-dependent, corresponding to 14 proteins (ANXA7, ANXA11, ANXA13, CAMK1, CALM1, CALR, CAPSL, CLCA4, CNN3, EFHC1, EFHD2, PEFLIN, TPM1, TPM4, WDR1, and VAT1) (Table 1). Regarding ZM, ANXA7, ANXA11, and WDR1 displayed increases in abundance, whereas VAT1, CAPSL, and CNN3 were decreased. Concerning QM, CNN3, EFHC1, EFHD2, PEFLIN, CAMK1, CALR, NIPSNAP1, and TPM1 increased in abundance whilst CALM1, TPM4 CLCA4, and ANXA13 showed a decrease (Table 1, last column).

### 3.3. Shared Protein Identity between the Two Species

Over and above the differences, identical proteins were found in ZM and QM. Their abundance after exposure to cadmium varied either in the same direction (12 proteins) or in opposite directions (10 proteins, Table 2).

Among the proteins with similar variations, increases in abundance were the most numerous, namely retinal dehydrogenase 2, cystathionine gamma lyase, eukaryotic translation initiation factor 3 subunit F, filamin A, heat shock protein 60, proliferation-associated protein 2G4, sulfotransferase family cytosolic 1A member 1, and phosphoenolpyruvate carboxykinase, partial. On the other hand, common decreases in abundance concerned 60kDa neurofilament protein, major vault protein, protein disulfite isomerase, and tubulin subunits.

The proteins showing an increase in abundance in QM and a decrease in ZM were mitochondrial ATP synthase delta chain, ATP-dependent RNA helicase DDX3X, far upstream element-binding protein 1, and mitochondria-eating protein. Finally, the proteins showing an increase in abundance in ZM and a decrease in QM were cytosolic non-specific dipeptidase, calponin homolog protein unc-87, maleylacetoacetate isomerase, cytoplasmic tryptophanyl-tRNA synthetase, cytosolic [GTP] phosphoenolpyruvate carboxykinase, and tubulin beta, partial. The proteins referred to as partial correspond to DAPs exhibiting a lower MW than expected.

### 3.4. Evidence of Short Proteoforms

It is noteworthy that in both species, some of the identified DAPs had a much lower molecular mass than expected, suggesting truncated proteins or alternative splicing events. It remains unclear whether the proteoforms have any other non-canonical functions [19]. Shorter proteoforms correspond mainly to cytoskeleton components such as alpha and beta tubulin subunits or filamin. Cadmium significantly impacts cytoskeleton elements which can impair cytoskeleton organisation [20]. Examples of cytoskeleton short proteoforms are presented in Figure S6.

**Figure 4:**
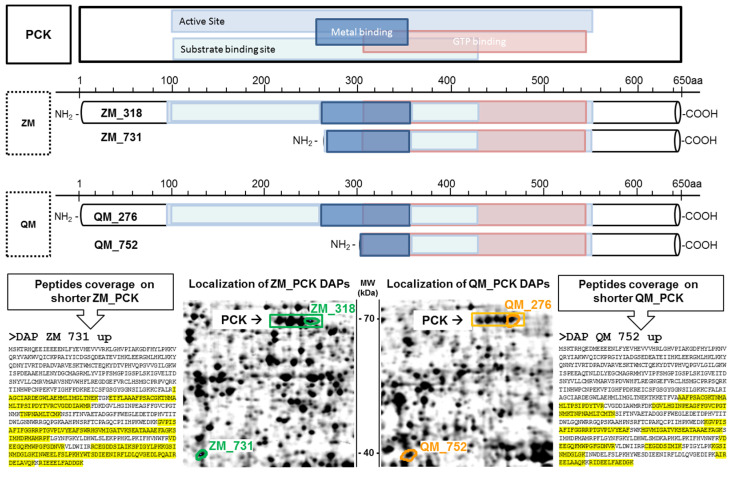
Shorter phosphoenolpyruvate kinase enzyme (PCK) proteoforms are more abundant after cadmium exposure. Structure of full-length PCK and shorter proteoforms of quagga (QM) and zebra (ZM) mussels. Top: PCK functional domains (NCBI CD batch search). Bottom: DAP localisation on 2DE gels; sequence coverage is highlighted in yellow.

Other shorter proteoforms are related to the phosphoenolpyruvate kinase enzyme (PCK). DAP QM 752 and DAP ZM 731 are approximately 40 kDa PCK proteoforms, while the standard PCK weighs around 70 kDa. Two DAPs corresponding to the full-length protein have also been identified, namely DAP QM 276 and DAP ZM 318. The presence of the shorter forms was confirmed through at least two gel excisions. MS data analysis using the Xtandem! pipeline showed that the shorter forms correspond to the second part of the enzyme in both species. It also confirmed a full sequence coverage for the 70 kDa proteoforms (Figure 4). The missing part in short proteoforms includes the active site, suggesting loss of enzyme activity after a proteolytic cleavage. The two truncated forms of PCK start at the metal binding site of the protein, but their formation cannot be explained, except that the PCK cofactor is the divalent ion manganese. It is possible for cadmium to replace manganese or disrupt its function. At least 2 PCK isoforms have been described, one cytosolic (PCK1) and the other mitochondrial (PCK2). They differ mainly in the first part of their sequence, so we could not establish whether the 40 kDa proteoforms originate from a cytosolic or mitochondrial PCK.

**Figure 5:**
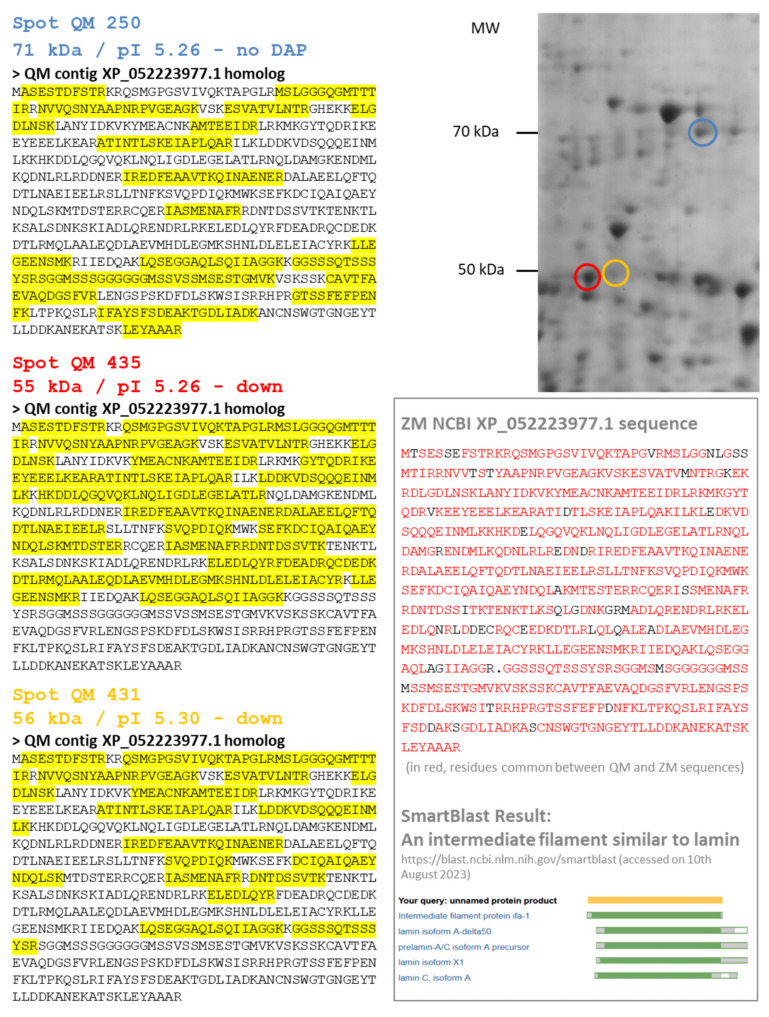
Several proteoforms for one protein: the case of a 60 kDa neurofilament protein-like. In quagga mussel (QM), several spots were identified using homology with the zebra mussel (ZM) XP_052223977.1 sequence: Spots 250, 435 and 431. Spot 250 is a 71 kDa protein and probably the precursor form of the 60 kDa neurofilament protein (blue circle on the 2DE gel). It presented no variation after cadmium exposure. Spots 435 and 431 are shorter sequences of 55 kDa (red and orange circles on the 2DE gel, respectively), corresponding to the first part of the XP_052223977.1 sequence (see MS peptide coverage highlighted in yellow). The XP_052223977.1 sequence of the 60 kDa neurofilament protein-like of ZM is presented in the black box, with the NCBI Smartblast result showing its alignment with lamins.

A third type of a short proteoform corresponding to XP_052223977.1 and named 60 kDa neurofilament protein-like (alias LMNA, lamin-A) was observed. Both species exhibited a decrease in abundance for the DAPs which were identified as 60 kDa neurofilament protein-like. However, these DAPs did not exhibit the same molecular weight (approximatively 70 kDa for DAP ZM 319 and 55 kDa for DAPs QM 431 and 435). In QM gels, we also identified a spot (QM 250) which remains constant and corresponds to the 70 kDa form of the 60 kDa neurofilament protein-like (Figure 5). In fact, XP_052223977.1 is a 70 kDa lamin precursor of intermediate filament. Its cleavage leads to lamin A/C, a component of the nuclear lamina. Alternative splicing events may explain the existence of DAPs with molecular weights less than 70 kDa.

A fourth type of short proteoform corresponds to an approximatively 50 kDa HSP70 (Figure S7). Detected solely in QM, its abundance is increased in response to cadmium exposure. Stable fragments of HSP70 have previously been evidenced in other studies, particularly in gills of blue mussels after acute heat stress [21]. It is unclear whether these truncated HSP70 proteoforms play a role during stressful conditions or whether they are indicative of an increasing level of intracellular proteolysis, which is more likely given the presence of other short proteoforms in QM gills exposed to cadmium.

**Figure 6:**
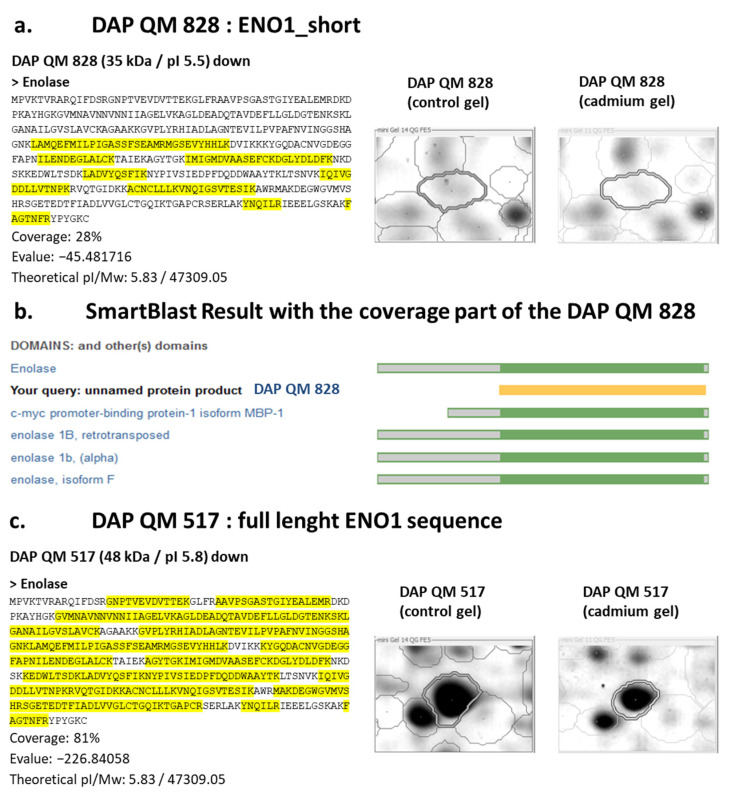
A quagga mussel (QM) shorter enolase proteoform is a putative c-myc promoter-binding protein-1 isoform. (**a**) 37 kDa DAP QM 828 was identified as enolase (ENO1) and lacks the first part of the enzyme; (**b**) the shorter enolase is highly similar to the c-myc promoter-binding protein-1 isoform (MBP-1); (**c**) 48 kDa DAP QM 517 is also identified as enolase; coverage (highlighted in yellow) on the full-length sequence confirms this DAP correspond to the whole enzyme.

Finally, a 37 kDa DAP QM 828 was identified as enolase (ENO1) whereas the canonical enzyme has a molecular mass of 48 kDa. MS analysis showed that this DAP lacks the first part of the ENO-1 (Figure 6a).

Furthermore, the 37 kDa DAP QM 828 is highly similar to an alternative splicing isoform called c-myc promoter-binding protein-1 isoform (MBP-1), a tumour suppressor (Figure 6b). Using an alternative start codon, the ENO1 gene can produce a truncated 37 kDa protein, mainly localised in the nucleus. MBP-1 lacks the first 96 amino acids present in ENO1 and its primary function is to bind to the c-myc transcription factor and suppress its activity [19]. Further experimentations are required to clarify the synthesis of the short ENO1 proteoform and its potential function. It is worth noting that after cadmium exposure, another DAP was also identified as ENO1, DAP QM 517. This proteoform was of 48 kDa, and exhibited complete coverage of the enolase sequence, indicating that the DAP QM 517 corresponds to the entire enzyme (Figure 6c). Finally, it should be highlighted that enolase, regardless of its form, was found to undergo modifications upon exposure to cadmium stress, as shown in numerous studies in both vertebrates and invertebrates [22,23,24,25].

## 4. Discussion

The objective of this work was to compare the responses of two sister species (*Dreissena* sp.) under stress by carrying out quantitative comparisons of the gill tissue at protein level. In theory, proteomics makes it possible to compare all the proteins in a tissue, but in practice, technical limitations reduce the number of actually compared proteins. Without being exhaustive, the extraction and separation conditions used here favour soluble proteins and offer better resolution for proteins with isoelectric points between four and seven and molecular weights between 10 and 100 kDa. In addition, the 2DE approach mainly reveals the most abundant proteins in the tissue and, as a result, modifications of proteins involved in basic cellular metabolism as well as in cellular trafficking and organisation were easily detected. However, gel-based proteomic analysis has the advantage of being an open approach capable of highlighting post-translational modifications (PTMs) which are very important in cellular homeostasis [26]. Regarding PTMs, not all of them are directly detectable on 2D gels, but only those that result in a sufficiently large change in molecular weight and/or isoelectric point to appear distinct from the main proteoform of the identified protein. It should be noted that several DAPs share the same identification, which means that first, the total number of identified proteins is much lower than the number of modified proteoforms, and second that post-transcriptional and/or post-translational modifications were involved in the response following cadmium exposure. Interestingly, the extent of the response differed between the sister species with 5.6% of varying proteoforms for ZM and 9.4% for QM, a difference mainly explained by a doubling of under-expressed proteoforms in QM. Therefore, proteomic analysis revealed a higher effect of cadmium on QM gill proteome than on ZM gill one, consistent with the higher accumulation of the metal in QM tissues at the end of the experiment.

### 4.1. DAP Classification and Protein Identification

The review of the literature regarding the identified proteins offers supporting information to comprehend our observations. Therefore, when interpreting the results, we considered the effect of cadmium, the identity of the protein, and the likelihood of it being a modified proteoform. Considering the scarcity of information available on bivalves in general and in mussels more specifically, we incorporated homology-based information as it pertains to humans and used GeneCards abbreviations for the database search. Then, the data were processed utilizing previously established bibliographic records on the effects of cadmium and our understanding of the bivalve physiology [20,27,28,29,30,31,32].

In some instances, there was no correspondence between the mussel protein and a human protein, which led us to assign a default GeneCards abbreviation. This was the scenario for ZM 641 DAP, which was recognised as an arginine kinase responsible for reversibly catalysing the transfer of phosphate between ATP and the phosphogen arginine phosphate in invertebrates, similar to creatine kinase (CKB) and the phosphogen creatine phosphate found in vertebrates. Arginine kinase and creatine kinase have a crucial function in the energy transduction process of tissues with high and varying energy needs, such as those in mussel gills [15,33]. It is noteworthy that this tissue is involved in osmoregulation in bivalves, where free amino acids, including taurine, aspartate, alanine, glutamate, glycine, and arginine, act as organic osmolytes [34,35]. Likewise, we assigned the abbreviation LDHB1 (lactate dehydrogenase) to DAB ZM 20062, identified as octopine dehydrogenase (EC 1.5.1.11), for which the KEGG pathway is also related to the metabolism of arginine and proline [36]. In all cases, the interpretation of the results was based solely on the knowledge of the identified protein and not on any arbitrary correspondence. Not all of the proteins identified are discussed in the text. However, for each protein, a concise summary of information gathered from GeneCards is provided in Supplementary Materials (https://www.genecards.org, accessed on 10 August 2023), also including bibliographic references related to cadmium exposure (Table S5).

### 4.2. Proteome Responses to Cadmium Exposure

In aquatic organisms, cadmium can be absorbed via digestion or internalised by the gills, borrowing calcium or other metal transporters. At the cellular level, cadmium has pleiotropic toxic effects that alter the homeostasis of calcium and essential divalent metals, the redox state with an overproduction of reactive oxygen species leading to the oxidation of macromolecules such as proteins, lipids, and DNA along with an impairment of DNA repair mechanisms, thus activating apoptosis and cell death [27,30,31].

At the molecular level, the cadmium cation shares chemical properties with other divalent ions (e.g., Zn^2+^, Fe^2+^, Ca^2+^) allowing for it to cross biological barriers and accumulate in tissues. In cells, the substitution of an essential metal ion, e.g., zinc or manganese, can occur during the folding process of nascent polypeptide chains, which may have an impact on their biological activity. Moreover, cadmium interacts with sulfhydryl groups and can lead to the alteration of proteins containing thiol groups and the depletion of reduced glutathione [37,38,39].

Oxidative stress is a major mechanism leading to cadmium-induced tissue damages. By competing with essential metal ions (e.g., Cu^2+^, Fe^2+^, Mn^2+^, Zn^2+^), cadmium increases cellular concentrations of free metal capable of a Fenton-type reaction and, by interacting with reduced glutathione, it decreases antioxidant defences, thereby promoting the oxidative action of reactive oxygen species [20,26,27,28,29,30,31,32,40,41].

**Figure 7:**
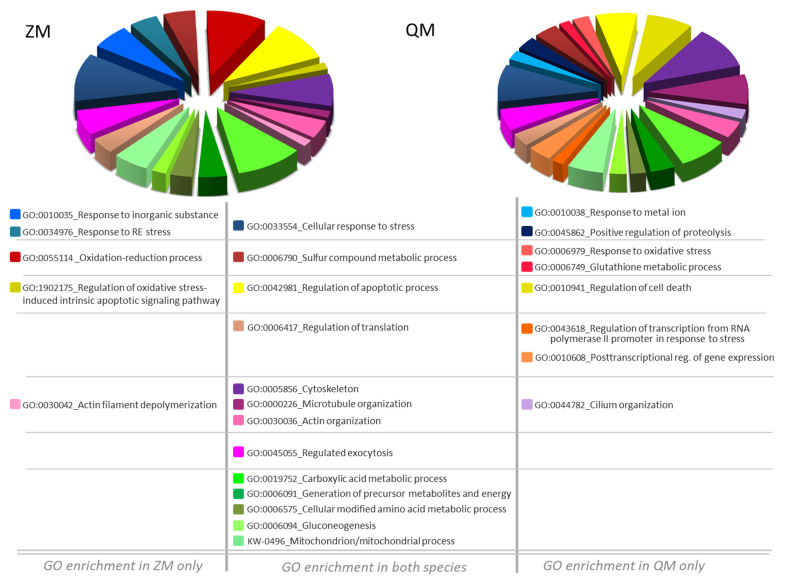
Pie Chart Diagram of GO biological process enrichment after Dreissena exposure to cadmium. Genome Ontology (GO) enrichment analysis of Cd induced DAPs-related biological processes. Allocation of biological processes is detailed in Table S4. Left, zebra mussel (ZM) and right, quagga mussel (QM).

The DAPs identified in both species mirror the aforementioned differential effects of the toxicant. Analysis of GO enrichment revealed strategies shared by and unique to both species in response to Cd exposure. In short, both species share the following cellular processes: induction of the cellular response to stress (GO:0033554), involvement of components of sulphur metabolism (GO:0006790), regulation of the apoptotic process (GO:0042981) and of translation (GO:0006417), changes in the generation of precursor metabolites and energy (GO:0006091), changes in the organisation of the cytoskeleton (GO:0000226, for microtubules; GO:0030036 for actin), and enhancement of exocytosis processes (GO:0045055) (Table S4; Figure 7).

Noticeably, in ZM only, GO enrichment analysis highlighted a response to inorganic substance (GO:0010035), reticulum endoplasmic stress (GO:0034976) and marked changes in proteins involved in the oxidation–reduction process (GO:00055114) with regulation of oxidative stress-induced intrinsic apoptotic signalling pathway (GO:1902175) (Figure 7). Differently and remarkably, in QM only, GO enrichment analysis pointed out a response to metal ion (GO:0010038) with positive regulation of proteolysis (GO:0045862). In this species, there is a notably greater number of altered cellular processes: response to oxidative stress (GO:0006979), glutathione metabolic process (GO:0006749), regulation of cell death (GO:0010941), regulation of transcription from RNA polymerase II promoter in response to stress (GO:0043618), post-transcriptional regulation of gene expression (GO:0010608), and cilium organisation (GO:0044782) (Figure 7).

### 4.3. Overlapping Modifications between ZM and QM

#### 4.3.1. Chaperon, Folding, and Degradation

Components of the chaperone, processing, folding, and degradation category mediate the correct folding of newly synthetised proteins and the detoxification of damaged or misfolded ones [29,42]. Activation of the cell response to stress was detected in both species. Indeed, several HSPs underwent abundance modifications in either both species (Heat shock proteins 70, HSP70; mitochondrial Heat shock protein family D member 1, HSPD1) or in one of the two species (ZM: HSPB1, DNAJB11, HSP90AA1, HSP90B1, and QM: HSPA5; for details, see Table 1B and Table S5), showing the set-up of a process to withstand cadmium-induced stress.

HSP70s play a critical role as protein chaperones, protecting the proteome from da-mages and cells from injury. They are involved in the folding of nascent or misfolded proteins, and in triggering the degradation of misfolded proteins if a proper structure cannot be restored. They also chaperone the association or dissociation of multi-protein complexes. Under oxidative stress, HSP70 additionally promotes the 20S proteasome-driven recycling of oxidatively damaged proteins [43,44].

HSPD1, alias hsp60 or 60 kDa heat shock protein, also displayed an increased abundance in both species. This chaperone is involved in the import of proteins into the mitochondrial matrix and, along with Hsp10, enables their correct folding. In case of mitochondrial stress, HSPD1 protects proteins from misfolding and promotes the proper refolding of the unfolded peptides produced during stress [45]. The induction of a mitochondrial HSP response is consistent with the fact that mitochondria are known targets of cadmium [32,45,46,47,48].

A decrease in abundance of the beta subunit of prolyl 4-hydroxylase (P4HB) has been observed in both species. P4HB is the ER most abundant disulfide isomerase and is involved in the synthesis and rearrangement of disulfide bonds in nascent and misfolded proteins. Moreover, it may act as a molecular chaperone amid ER stress. This function is closely linked to its concentration. At high concentrations, P4HB prevents the aggregation of unfolded proteins, whereas at low concentrations, it promotes aggregation [49]. This may be relevant here as cadmium exposure decreases its abundance.

#### 4.3.2. Antioxidant Response and Detoxification

Modification of antioxidant and biotransformation systems is a shared trait between ZM and QM. For instance, cadmium exposure elicited differential regulation of superoxide dismutases (SOD) and gluthatione-S-transferases (GST) (ZM: SOD2; QM SOD1, GST, GSTP1; for details, see Table 1B and Table S5). This response was expected due to cadmium’s properties and its ability to indirectly cause oxidative stress [22,24,31,40,50,51,52,53,54,55,56,57,58,59,60].

In particular, aldehyde dehydrogenase 1 family member A2 (ALDH1A2) abundance increased after cadmium exposure in both *Dreissena* species. Also known as retinal dehydrogenase 2, ALDH1A2 belongs to the aldehyde dehydrogenase family. ALDH1A2 converts retinaldehyde to retinoic acid, which plays a critical role in several cellular and molecular processes such as development, immunity, cell proliferation, or apoptosis by modulating retinoid signalling pathway. This cytosolic enzyme oxidises retinal and aliphatic aldehydes into the corresponding carboxylic acid, including 4-hydroxy-2-nonenal, a typical product of lipoperoxidation. ALDH1A2 could therefore reduce oxidative stress [61,62].

Furthermore, the GO term “sulphur compound metabolic process” is relevant to many DAPs in the antioxidant and detoxication category, e.g., previously cited GSTs, or glutaredoxin-3 (GLRX3) and bifunctional 3′-phosphoadenosine 5′-phosphosulfate synthase 1 (PAPSS1) for ZM and glutathione reductase (GSR) or glutathione synthetase (GSS) for QM (for details, see Table 1C, DAPs in bold). This is consistent with the presence of amino acids such as methionine or cysteine, or glutathione among the sulphur compounds. Moreover, sulfotransferase family 1A member 1 (SULT1A1) and cystathionine gamma-lyase (CTH) were upregulated by cadmium exposure. SULT1A1 is a sulfotransferase implicated in sulphate conjugation using 3′-phospho-5′-adenylyl sulphate as a sulphate donor. Sulfonation enhances the hydrophilicity of the resultant conjugate, facilitating excretion. Regarding CTH, this enzyme is responsible for catalysing the final step in cystathione conversion to L-cysteine. Part of cysteine is used to synthesise glutathione, a key component in regulating the intracellular redox state. CTH has the ability to produce persulfides and polysulfides from cysteine resulting in products that may have multiple functions. By sulfhydrating the specific cysteine residue of target proteins, CTH can regulate their function. Additionally, persulfides and polysulfides produced in a non-specific manner are known to capture heavy metals such as cadmium, thereby facilitating their excretion. Finally, CTH can generate hydrogen sulphide (H_2_S) from two cysteines. H_2_S can prevent cell damage in the same way as persulfides while also acting as a gazotransmitter involved in several signalling pathways. Studies on frogs or chironoma showed an increase in CTH gene expression when exposed to heavy metals [38,39]. Furthermore, in myoblast cells, Zhang and colleagues proposed a pathway underlying the involvement of CTH and the produced H_2_S in cell protection against Cd-induced cell death [63]. The increased abundance of cystathionine γ-lyase is related to its critical position between glutathione synthesis and the integrated stress response, which helps to protect tissues from ER stress [64].

#### 4.3.3. Translation Regulation

Control of protein synthesis is a key determinant of cell growth. Even slight modifications to the proteins involved in translation can alter protein synthesis, which in turn can change the fate of a normal cell, leading it toward a tumorous or apoptotic state [65]. Several DAPs involved in regulating translation were identified in response to cadmium exposure: EIF5A, EEF2 (ZM), and EIF4A1, EIF4B (QM, for details, see Table 1 and Table S4). Additionally, two DAPs, namely eukaryotic translation initiation factor 3 subunit F (EIF3F) and proliferation-associated protein 2G4 (PA2G4), were detected in both species with increased abundance.

EIF3F is a noncore subunit of the eukaryotic translation initiation factor 3 complex, necessary for multiple phases of the protein synthesis initiation. The EIF3 complex is specifically involved in the translation of transcripts encoding proteins linked with cell proliferation and apoptosis. The composition of the EIF3 complex can differ, and variants exhibit cytosolic and/or nuclear localisation, suggesting moonlighting functions. The occurrence of EIF3F has been associated with the inhibition of translation and, in turn, proposed as a negative regulator of cell growth and proliferation [65]. Lastly, the EIF3 complex has been advanced as a potential biomarker of cadmium exposure in a cell or an organism [66].

By being involved in transcriptional and translational regulations as well as ribosome assembly, PA2G4 also plays a role in regulating cell growth and proliferation. For example, in the cytoplasm, PA2G4 associates with mature ribosomes and functions as an inhibitor of eukaryotic initiation factor 2α (eIF2α), thus blocking translation. In humans, two splicing variants with opposite functions have been described: the long PA2G4, which suppresses apoptosis and promotes cell growth, acting as an oncogene, and the short one, which functions as a tumour suppressor, promoting cell differentiation and suppressing proliferation. No such splicing has been described in *Dreissena* sp., and when we aligned the human and *Dreissena* sequences, it seemed likely that the two identified DAPs correspond to the full-length protein [67].

#### 4.3.4. Energy Metabolism and Mitochondrial Alterations

Cadmium exposure affected carboxylic acid, amino acid metabolic and mitochondrial processes, including gluconeogenesis and generation of precursor metabolites and energy, all common to both species (for details, see Figure 3, Table 1D). Setting up molecular responses to combat metal toxicity requires energy. Organisms must increase their energy production by using the available carbohydrate reserves and by adjusting the function of several metabolic pathways [68]. In addition, proteins involved in metabolic pathways may themselves be targeted by cadmium, leading to additional perturbations.

No common DAPs were identified between species. The carboxylic acid metabolic process (GO:0019752) appeared to be the main response with regard to the use of carbohydrate reserve and modulation of metabolic pathways including the glycolytic and the pentose phosphate pathways, amino acids metabolism, gluconeogenesis, and within mitochondria, the TCA cycle and the synthesis of ATP via oxidative phosphorylation.

Examining the subcellular localisation of the identified DAPs reveals a noteworthy proportion located in the mitochondria. Cadmium is a well-documented mitochondrial toxin, accumulating within these organelles in vertebrate and invertebrate organisms, including bivalves [45,46,47,48]. This accumulation results in mitochondrial uncoupling, impaired ATP synthesis, and cellular energy imbalance [69,70,71]. Cadmium exposure may induce, in fine, mitochondrial swelling and provoke mitochondrial-dependent apoptosis [20,32].

#### 4.3.5. Regulation of Apoptotic Process

The GO term “regulation of apoptotic process” is conserved between the two *Dreissena* species. As mentioned above, cadmium targets mitochondria and can induce apoptosis [27,30,72]. Interestingly, Joseph and colleagues reported an intricate connection between cadmium and apoptosis in cell culture studies. Cells surviving cadmium exposure could become resistant to cadmium-induced apoptosis. This could promote the survival and proliferation of cell with DNA damages, potentially leading to carcinogenic transformation [27]. These complex phenomena may explain the high representation of DAPs pertaining to the GO term “regulation of apoptotic process” among the identified DAPs. This is especially true for those that are common to both species, including P4HB, ALDH1A2, and CTH, as well as for the PA2G4, and 60 kDa neurofilament protein (LMNA) presented in the next section regarding the cytoskeleton.

#### 4.3.6. Cytoskeleton Modifications

Cadmium is a well-known stressor of the cytoskeleton, resulting in oxidative damage to its components and the disruption of its organisation [20,24,47,73,74,75,76,77,78,79,80,81].

In this study, a significant number of DAPs corresponding to components of the cytoskeleton (ZM: 24.7%; QM: 32.4%) were identified. These are mainly elements associated with microtubules and the beta and alpha subunits of tubulin itself. In both species, some identified DAPs exhibited much lower molecular mass than expected, suggesting fragmentation of alpha and beta tubulin subunits, filamin (FLNA), or LMNA. Regarding LMNA, in a proteomic study undertaken on freshwater mollusk *Lymnaea stagnalis* to investigate the molecular effects of nerve lesion, Perlson and collaborators identified cleavage products of intermediate filaments termed retrograde protein of 51 kDa (RGP51) which may perform both structural and signalling functions in retrograde injury signalling after cytoskeleton collapse [82]. Comparison of the MS/MS files indicated that 55 kDa LMNA could be consistent with the RGP51 sequence. In cadmium-exposed mussels, cleavages and modifications of intermediate filament abundance may participate to such a mechanism, i.e., rearrangement of cytoskeleton to redirect cell molecular trafficking under toxic pressure.

#### 4.3.7. Exocytosis

Many DAPs involved in cellular trafficking, membrane endocytosis, and exocytosis were detected (16 for ZM, 17 for QM) with the major vault protein (MVP) TUBB, FLNA, and LMNA, in common. Interestingly, MVP was detected with a decreased abundance in both species. Major vault proteins are the main components of Vault ribonucleoparticles. These barrel-shaped structures with an internal cavity participate in nucleo-cytoplasmic trafficking and can shuttle between the nucleoplasm and the cytoplasm even though Vaults are mainly cytosolic. Vaults act as scaffolds for proteins involved in signal transduction in multiple kinase signalling pathways [83]. Vaults can export DNA-damaging drugs, and their connection with multidrug resistance in cancer through the exocytosis of drugs has been established [83]. Moreover, MVP has been discovered on the surface of cancer cells, unlike in normal cells. It is believed that this extracellular variant is linked with cancer progression [83]. In the context of our findings, it is worth noting that MVP is cytoprotective, and downregulation of MVP has been demonstrated to increase sensitivity to cell death [84].

As a conclusion, the cellular-level disruption of the cytoskeleton and trafficking can be put into perspective with branchial physiology. Several histological studies have explored the effects of cadmium on bivalve gills. The presence of cadmium-rich inclusions, structural and functional changes in membrane trafficking, increased exocytosis linked with a greater number of mucus cells, hyperplasia of the gill lamellae, loss of the lateral cilia that hold the lamellae together, rupture of the endothelial cells, and even necrosis are among the toxic effects of cadmium [85].

### 4.4. Focus on Some Species-Specific Modifications

#### 4.4.1. DAPs That Display Common Identity but Opposite Direction of Variation

In our study, several proteins exhibited an increase in abundance in ZM and a decrease in QM, i.e., the far upstream element-binding protein 1 (FUBP1), the ATP-dependent RNA helicase (DDX3X), and the spermatogenesis associated 18 (SPATA18).

FUBP1 is a single-stranded DNA-binding protein that binds to multiple DNA elements, including the far upstream element (FUSE). FBP1 interacts with p53, functions as a regulator of p53-regulatory proteins, and suppresses p53 transactivation activity under radiation-induced cellular stress [86]. It also regulates the c-myc proto-oncogene, a master regulator of cell proliferation, differentiation, and apoptosis. FUBP1 regulates c-myc expression by binding to a single-stranded far-upstream element (FUSE) upstream of the c-myc promoter [87]. In ZM, the FUBP1 increase suggests a modulation via c-myc. However, in QM, we also identified a DAP capable of modifying c-myc: a short ENO highly similar to the c-myc promoter-binding protein-1 isoform (MBP-1), which shows a decreased abundance. MBP-1 binds to the c-myc transcription factor and suppresses its activity [19]. These observations are consistent with the activation of the c-jun Nterminal kinase (JNK) pathway under cadmium stress, resulting in increased expression of c-fos, c-jun, and c-myc [30]. In blood clam *Tegillarca granosa* gills, up-regulation of c-myc has also been observed after cadmium exposure, suggesting that c-myc regulates cadmium-induced stress response and detoxification in the bivalves [88].

DDX3X belongs to the DEAD-box helicase family and is involved in many aspects of RNA biology. Its functions include the transcription regulation of certain genes involved in cell cycle regulation, signalling pathways, or cell adhesion. It is also implicated in RNA splicing and export to the cytoplasm. Finally, DDX3X is involved in the initiation of both cap-dependent and cap-independent translation. In cancer cells, DDX3X was shown to globally repress protein synthesis while fostering the translation of specific RNAs by binding to the eIF4F complex. In the context of ER stress, DDX3X is required for the translation of ATF4, a key transcription factor in the integrated stress response. During cell stress, DDX3X plays a pivotal role in determining cell fate, as it is involved in two competing processes: it either contributes to the assembly of stress granules, which provide cytoprotection, or it can interact with NLRP3 (for NACHT, LRR and PYD domains containing protein 3) and trigger the inflammasome activation, leading to pyroptosis. The availability of DDX3X for either process acts as a central regulator of the balance between cell survival and death [89].

SPATA18, also known as the mitochondria-eating protein, is a key regulator of mitochondrial quality responsible for repairing or degrading damaged mitochondria. Its transcription is positively regulated by p53. When mitochondrial damage occurs, a process involving SPATA18 called mitochondrial eating protein-induced accumulation of lysosome-like organelles within mitochondria (MALM) is activated [90]. SPATA18 plays an essential role in MALM by opening a pore that enables lysosomal proteins to enter the mitochondrial matrix without altering the mitochondrial structure. The oxidised proteins are subsequently degraded by lysosomal hydrolases, resulting in increased ATP production. SPATA18 also participate in the mitophagy of damaged mitochondria. Recent studies have shown that SPATA18 silencing impedes mitophagy induction, resulting in the persistence of damaged mitochondria [91]. The opposite abundance variations of this protein suggest that mitochondrial repair processes were activated in ZM but not in QM.

Conversely, increases in proteins were observed in QM and decreases were noted in ZM, such as the cytoplasmic tryptophanyl-tRNA synthetase cytoplasmic (WARS1), manganese-dependent cytosolic non-specific dipeptidase (CNDP2), calponin 3 (CCN3) and maleylacetoacetate isomerase (GSTZ1).

WARS1 catalyses the ligation of tryptophan to its ARNt. In humans, WARS1 expression is induced by interferon γ and it is implicated in multiple physiopathological processes such as immunity or cancer [92]. Little is known about non-canonical functions of WARS in invertebrates, although modulation of WARS1 has already been described in scallops under stress [93]. Its increase in QM may reflect inflammation.

CNDP2 is ubiquitously expressed and highly conserved. CNDP2 exhibits a broad substrate specificity with a preference for cysteinyl–glycine, an intermediate metabolite in glutathione metabolism. In addition, in gastric cancer, CNDP2 may activate the p38 and JNK MAPK pathways, leading to cell apoptosis when highly expressed, or the ERK MAPK pathway, promoting cell proliferation when expressed at a low level [94]. If bivalve CNDP2 functions similarly, its reduction in ZM would suggest greater resistance to cadmium exposure, while its low abundance in QM would indicate an imbalance in favour of apoptosis.

CCN3 is a thin filament-associated protein implicated in actin bundling and actin filament dynamics, and it also contributes to actin filament stability. Decreases in calponin abundance have been documented in green mussels exposed to cadmium [22]. The opposite variation between ZM and QM is consistent with the distinct modifications of the actin cytoskeleton observed in these two species.

GSTZ1, also known as soluble glutathione S-transferase, is a multifunctional enzyme. Its main activity is linked to the phenylalanine/tyrosine degradation pathway with the ability to convert maleylacetoacetate into fumarylacetoacetate. GSTZ1, as a biotransformation enzyme, can also conjugate GSH, although this activity is deemed minor. A third function, i.e., glutathione peroxidase, has been described. GSTZ1 is considered as a sign of cadmium-induced stress [45]. The observed abundance variations, regardless of their direction, support GSTZ1 as a potential biomarker.

#### 4.4.2. ZM-Specific Modifications

ZM-specific proteome modifications in response to cadmium exposure can be illustrated by two GO terms, i.e., “Response to endoplasmic reticulum stress” and “Actin filament depolymerisation”. The GO term Response to endoplasmic reticulum stress was evidenced only in ZM with a group of 7 DAPs including dnaJ homolog subfamily B member 11 (DNAJB11), endoplasmin (HSP90B1), small heat shock protein p36 (HSPB1), transitional endoplasmic reticulum ATPase (VCP), and ubiquitin-fold modifier-conjugating enzyme 1 (UFC1). In addition to its ability to bind to thiol groups of proteins, cadmium can mimic calcium and interfere with its signalling pathway [20]. Disruption of calcium homeostasis in the endoplasmic reticulum can cause protein misfolding and result in ER stress [95]. Cellular response to stress is present in both species, but ZMs seem to develop stronger defence mechanisms to face cadmium.

As previously mentioned, actin is a target of oxidative stress resulting from cadmium exposure [73,74]. The cytoskeleton may deeply affect the modified dynamics of actin polymerisation/depolymerisation and potentially disrupt the focal adhesion contacts [20]. In ZM, several DAPs are actin-related proteins, including twinfilin actin binding protein 1 (TWF1), cofilin-1 (CFL1), and WD repeat-containing protein 1 (WDR1). They all play a role in cytoskeletal organisation in epithelial cells such as gills and exhibit an increased abundance in response to cadmium. TWF1 and CFL1 are actin-binding proteins that inhibit actin polymerisation. WDR1 alias actin-interacting protein 1 is regulator of actin-dependent processes increasing the stability of cortical actin in a calcium-dependent manner and, conversely, the disassembly of actin filaments in conjunction with cofilin family proteins [76]. Lewinska and colleagues showed that c-myc activation promotes cofilin-mediated remodelling of the actin cytoskeleton in response to oxidative DNA damage [96]. In ZM, increased abundances of TWF1 and CFL1 can be related to aforementioned modifications of c-myc regulation, implying that repair mechanisms are activated.

#### 4.4.3. QM-Specific Modifications

As already mentioned, in QM only, GO enrichment analysis indicated a response to metal ion with positive regulation of proteolysis and a notably greater number of altered cellular processes including regulation of cell death and cilium organisation (Figure 3, Table S4).

Cytoskeletal modifications are more pronounced in QM, with a larger number of modified proteins belonging to the microtubule organisation and specifically to the organisation of ciliated epithelial structures. These observations imply more significant effects of cadmium on the QM gills, resulting in marked histological and physiological alterations. In gills of the clam, *Meretrix meretrix*, cadmium leads to mitochondria-mediated apoptosis with induction of caspases and disorganised ciliary orientation with impairment of microtubules [48]. Consistent with these protein alterations, changes in the proteasome are also specifically observed in QM. The 26S proteasome is involved in the ATP-dependent degradation of ubiquitinated proteins. This multiprotein complex is formed by association of the 20S core particle with the 19S regulatory particle. It plays a key role in the maintenance of protein homeostasis by removing misfolded or damaged proteins, which could impair cellular functions, and by removing proteins whose functions are no longer required [44]. Under oxidative stress, damaged protein accumulation in the cell could partly be removed by proteasome activity. In QM only, several DAPs have been identified as subunits of the proteasome: 26S proteasome non-ATPase regulatory subunit 14 (PSMD14), 26S proteasome regulatory subunit 10B (PSMC6), proteasome subunits alpha type-2 (PSMA2), and proteasome subunit alpha type-4 (PSMA4). The PSMD14 alias Rpn11 is a deubiquitinating enzyme that belongs to the metalloenzyme JAMM family. PSMD14 is a deubiquitinating enzyme for proteins directed towards the proteasome. The enzyme is a metalloprotease that contains a Jab1/MPN metalloenzyme domain motif (JAMM) linked to the zinc ion, so cadmium may impair the zinc–JAMM interaction. PSMD14 plays a key role in maintaining proper mitochondrial function. Furthermore, PSMC6 is a member of the AAA proteins (for ATPases associated with diverse cellular activities proteins) and plays a role in deubiquitylation by unfolding polyubiquitinated proteins. This allows for their entrance into the catalytic zone of the proteasome, following DUB polyubiquitin chain cleavage. PSMA2 and PSMA4 are components of the 20S core proteasome complex. Oxidative stress favours the disassembly of the 26S proteasome complex into 20S and 19S particles and the degradation of oxidised proteins by the 20S core. Consequently, the 20S proteasome appears to be the major machinery involved in degradation of oxidised proteins in response to oxidative stress, even if degradation of damaged proteins can still occur by ubiquitin/ATP-dependent activity of the 26S proteasome [44]. Finally, this observation indicates that cadmium induces more proteolytic processes in QM than in ZM, as also supported by the GO enrichment positive regulation of proteolysis (GO:0045862) in QM. Significant enrichment of the cellular proteolysis process was also observed in clams following exposure to 50 μg·L^−1^ cadmium [24].

## 5. Synthesis

**Figure 8:**
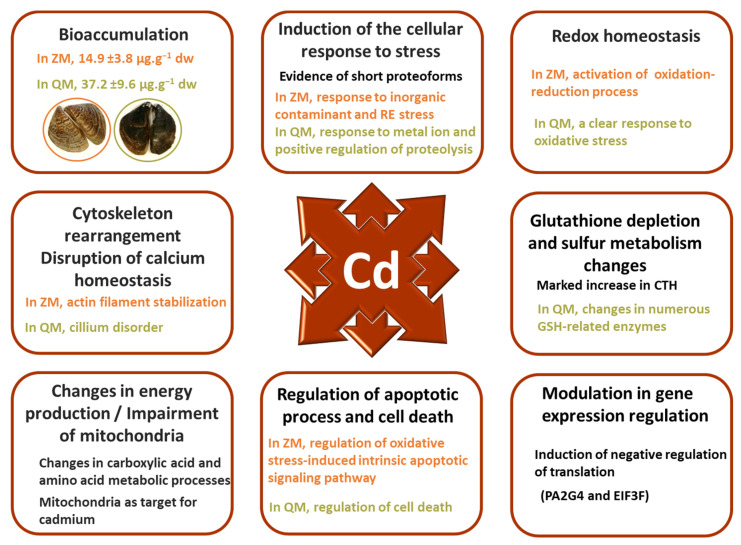
Modulations of Dreissena gill proteomes are consistent with Cd exposure but partly differ reflecting species-specific cellular strategies in fighting homeostasis rupture.

Overall, cadmium exposure in both species resulted in the development of resistance mechanisms to cadmium-induced apoptosis (Figure 8). Hence, cellular stress response activation was detected in both species. Regarding protein synthesis, we observed the induction of proteins involved in negative regulation of translation such as PA2G4 and EIF3F; the latter has already been described as a potential biomarker of cadmium exposure [66]. In parallel, marked HSPD1 and HSP70 increases were detected, indicating the activation of folding and degradation pathways. Moreover, a decreased abundance of P4HB, which promotes aggregation of unfolded proteins at low levels, is another indicator of ER-stress observed in both species. Common responses also relied on a specific antioxidant cell response against Cd-induced cell death, marked by increased levels of ALDH1A2, SULT1A, and CTH. CTH could be proposed as a potential biomarker of cadmium exposure due to its involvement in the production of H_2_S, which helps to prevent cell damage by capturing heavy metals. As expected, both species displayed changes in protein abundances implicated in metabolic pathways, including glycolytic and pentose phosphate pathways, amino acid metabolism, gluconeogenesis, TCA cycle, and oxidative phosphorylation. It is likely that these changes are related to an increased demand for energy.

Cadmium toxicity mainly affects the mitochondria, cytoskeleton, and calcium signalling, leading to oxidative stress. The modifications observed in the sister species are largely linked to these deleterious effects, but species-specific responses were detected. For example, the non-specific antioxidant response was stronger in ZM compared to QM. There is more evidence of cytoskeleton rearrangement and increases in cellular trafficking and membrane exocytosis in QM compared to ZM. These changes may be associated with more gill cilia damage in QM. The results also suggest an activation of the c-myc regulatory pathway in both species, although mediated differently. In QM, a decrease in a short ENO proteoform highly similar to the c-myc promoter-binding protein-1 isoform (MBP-1) was observed. In ZM, increased abundances of FUBP1, TWF1, and CFL1 indicate cofilin-mediated remodelling of the actin cytoskeleton promoted by c-myc activation in response to oxidative DNA damage. Opposite abundance variations of some proteins, including SPATA18, CNDP2, WARS1, CCN3 and GSTZ1, suggest a better activation of mitochondrial repair processes and a greater resistance to cadmium exposure in ZM than in QM, where inflammation and apoptotic processes appeared more evident. It should be noted that GSTZ1 variation is considered as a sign of cadmium-induced stress.

Interestingly, proteoforms with molecular weights significantly lower than those of the canonical proteins were observed. The presence of these proteoforms is more prominent in QM than in ZM. The question of a non-canonical function is raised for some of these short proteoforms.

Taken together, the findings of this work are in accordance with prior studies indicating that ZM populations have a greater ability to activate their defence mechanisms when facing contamination, while QM appear less able to adapt to coping with the contamination they are exposed to [97]. It is also noteworthy that biomarker assessment following a 7-day cadmium exposure (10 µg·L^−1^) revealed no response in QM while ZM showed variations [8]. This could be associated to the inability of QM to induce functional defence mechanisms rather than to a resistance to contamination. Indeed, similar responses were observed following Ni short-term exposure, and they were finally related to a lower tolerance of QM [98]. Cadmium can trigger both autophagy and apoptosis, thereby impacting cell viability [47,99]. The difference in cadmium tolerance between the two species may be explained by the formation of apoptotic degradosome vesicles in QM and a potential autophagic response in ZM. Finally, QMs appear to be less tolerant to high stress levels than ZMs, which display the ability to activate distinct cellular strategies to cope with homeostasis disruption in the event of contamination [98].

This work is in line with the known pleiotropic effects of cadmium. However, it also highlights species-specific responses with proteoforms identified solely in QM or ZM, or common proteoform exhibiting opposite variations of abundances. It would be of interest to further compare QM and ZM responses to heavy metal through multi-omics approaches.

## 6. Conclusions

A classic pollutant was used to highlight the difference in stress response between two mussel species used as sentinel in freshwater biomonitoring. Cadmium treatment induced significant proteomic alterations in the gills of quagga and zebra mussels that are consistent with the known effects of cadmium exposure. Overall, QM displayed higher cadmium accumulation and marked alterations in the gill proteome compared to ZM. Although sharing common features, the proteomic modification profiles exhibited significant differences. In particular, QM and ZM react to cadmium by implementing distinct defence and survival responses. This reinforces our view that the two species are not interchangeable in environmental biomonitoring, emphasising the importance of precise species identification.

## Figures and Tables

**Table 1:** Differentially abundant proteoforms identified in zebra (ZM) and quagga (QM) mussel gills after cadmium exposure.

**Part A. Chaperone/processing/folding/degradation (GO:0033554_Cellular response to stress in bold)**								
Sp.	label	abbrev.	Mr	pI	Var.	Identification	G.	Ca
ZM	44,479	COL12A1	83,379	5.6	2.07	collagen alpha-1(XII) chain		
ZM	346	**HSPA12A**	69,540	6.6	1.83	heat shock 70 kDa protein 12A		
ZM	645	**PDIA6**	44,461	5.6	1.68	protein disulfide-isomerase A6	1	
ZM	8514	NAPA	24,787	5.0	1.64	alpha-soluble NSF attachment protein	7	
ZM	901	TGFBI	34,177	7.4	1.52	transforming growth factor-beta-induced protein ig-h3		
ZM	434	**PPP5C**	56,199	6.0	1.49	serine/threonine-protein phosphatase 5	1	
ZM	2519	**HSPD1**	54,076	5.4	1.46	heat shock protein 60, mitochondrial	1; 6	
ZM	102,964	**UFC1**	23,929	6.8	1.39	ubiquitin-fold modifier-conjugating enzyme 1	1	
ZM	163	RRBP1	89,856	5.6	1.35	ribosome-binding protein 1		
ZM	984	**CA2**	29,793	6.1	1.34	carbonic anhydrase II	1	
ZM	817	HSPB1	38,223	6.5	1.28	small heat shock protein p36	6	
ZM	705	**DNAJB11**	42,109	5.7	1.26	dnaJ homolog subfamily B member 11	1	
ZM	211	**HSP90AA1**	85,659	5.1	0.78	heat shock protein 90	1; 6; 7	
ZM	170	MVP	89,818	5.7	0.74	major vault protein	7	
ZM	207	**VCP**	85,723	5.0	0.65	transitional endoplasmic reticulum ATPase	1; 6; 7	
ZM	159	**HSP90B1**	89,954	5.1	0.61	endoplasmin	1; 6	
ZM	655	**P4HB**	44,315	4.4	0.56	protein disulfide-isomerase	1; 6	
ZM	334	**STIP1**	73,026	5.8	0.55	stress-induced-phosphoprotein 1		
QM	583	CCT5	45,634	6.1	3.34	T-complex protein 1 subunit epsilon	4	
QM	382	**HSPA1**	58,954	5.4	1.81	heat shock protein 70, partial	1; 4; 6; 7	
QM	851	**PSMD14**	34,207	6.1	1.81	26S proteasome non-ATPase regulatory subunit 14	1; 7	
QM	673	**PSMC6**	41,340	6.2	1.71	26S proteasome regulatory subunit 10B	1	
QM	341	ATP6V1A	63,911	5.4	1.69	V-type proton ATPase catalytic subunit A		
QM	546	**HSPD1**	46,798	5.5	1.61	heat shock protein 60, mitochondrial	1; 6	
QM	1058	**PSMA4**	28,393	5.5	1.56	proteasome subunit alpha type-4	1	
QM	625	**CASP3**	43,567	4.8	1.55	caspase 3 like	1; 6	
QM	1066	YWHAB	28,086	4.9	1.47	14-3-3 protein 1		
QM	839	PEPD	34,135	5.7	0.74	proline iminopeptidase	3	
QM	424	CCT4	57,096	6.2	0.70	T-complex protein 1 subunit delta	4	
QM	1026	CTSL	28,816	4.6	0.69	cathepsin L		
QM	291	**HSPA1**	68,940	5.4	0.67	heat shock protein 70	1; 4; 6; 7	
QM	1137	**PSMA2**	27,167	5.5	0.66	proteasome subunit alpha type-2	1; 7	
QM	437	ATP6V1B1	55,137	5.7	0.65	V-type proton ATPase subunit B	3	
QM	299	**HSPA5**	66,677	5.2	0.62	Endoplasmic reticulum chaperone BiP GRP78	1	
QM	620	CTSD	43,053	4.6	0.61	cathepsin D	6; 7	
QM	4174	**PACRG**	27,552	7.0	0.59	parkin coregulated gene protein homolog	1	
QM	748	**P4HB**	38,485	4.7	0.58	protein disulfide-isomerase	1; 6	
QM	100	MVP	94,623	5.7	0.56	major vault protein	7	
QM	985	**YWHAE**	29,752	4.7	0.53	14-3-3 protein epsilon	1	
QM	151	MVP	88,509	5.7	0.52	major vault protein, partial	7	
**Part B. Calcium and cytoskeleton alterations (GO:0005856 Cytoskeleton in bold)**								
Sp.	label	abbrev.	Mr	pI	Var.	Identification	G.	Ca
ZM	191	**FLNA**	85,827	6.0	1.63	filamin-A, partial	4; 6; 7	
ZM	834	**PPP1CA**	38,250	5.8	1.63	serine/threonine-protein phosphatase alpha-2 isoform	1; 4; 6	
ZM	244	ANXA7	78,074	7.6	1.62	annexin A7	7	Ca
ZM	597	**PRKAR2B**	45,791	4.7	1.51	cAMP-dependent protein kinase type II regulatory subunit	3; 4	
ZM	722	**NME7**	41,220	6.3	1.50	nucleoside diphosphate kinase 7	4	
ZM	83,183	IFI44	47,224	6.6	1.45	microtubule-associated protein 44		
ZM	932	ANXA11	33,143	5.8	1.43	annexin A11	4; 7	Ca
ZM	82,002	**WDR1**	64,919	6.6	1.43	WD repeat-containing protein 1	4; 7	Ca
ZM	723	**TWF1**	41,814	5.8	1.40	twinfilin-1	4	
ZM	766	SPAG6	40,347	5.9	1.39	sperm-associated antigen 6	4	
ZM	679	DNPEP	42,701	6.3	1.29	aspartyl aminopeptidase	7	
ZM	1334	**CFL1**	13,142	4.6	1.25	cofilin	4; 6	
ZM	550	SNX6	47,482	5.7	1.22	sorting nexin-6	6	
ZM	1039	**TUBB**	28,478	5.3	0.80	tubulin beta, partial	4; 7	
ZM	610	VAT1	45,137	5.7	0.73	synaptic vesicle membrane protein VAT-1 homolog	7	Ca
ZM	20,235	**TUBA1A**	46,039	5.8	0.68	tubulin alpha-1A chain	4; 7	
ZM	1173	CAPS	25,562	5.4	0.68	calcyphosin protein		Ca
ZM	110	**FLNA**	94,619	5.7	0.65	filamin A_B_C partial	4; 6; 7	
ZM	9159	**CNN3**	22,873	5.1	0.56	calponin homolog, protein unc-87	4	Ca
ZM	319	**LMNA**	75,067	5.2	0.55	60 kDa neurofilament protein-like	1; 4; 5; 6; 7	Ca
QM	182	CFAP58	83,075	6.6	3.75	cilia- and flagella-associated protein 58		
QM	177	PEFLIN	83,987	6.9	2.82	peflin		Ca
QM	1116	EFHD2	27,319	5.5	1.89	EF-hand domain-containing protein D2		Ca
QM	632	**CROCC**	43,108	6.1	1.87	rootletin	4	
QM	992	**CNN3**	29,595	5.3	1.70	calponin homolog, protein unc-87	4	Ca
QM	188	**FLNA**	83,094	5.9	1.65	filamin-A, partial	4; 6; 7	
QM	198	**VCL**	80,926	5.6	1.64	vinculin	4; 7	
QM	1119	**CALM3**	27,412	5.2	1.62	calmodulin protein 3		Ca
QM	718	CALR	39,142	4.8	1.60	calreticulin	1; 4; 5; 6	Ca
QM	508	**TUBB4B**	48,029	5.4	1.59	tubulin beta-4B chain	4	
QM	495	SYNE2	49,936	5.6	1.53	nesprin-2	4	
QM	1090	**EFHC1**	27,602	5.2	1.52	EF-hand calcium-binding domain-containing protein 1	4	Ca
QM	926	**TBCB**	31,668	4.8	1.42	tubulin-folding cofactor B	4	
QM	1219	**TMSB10**	27,111	5.3	1.39	thymosin beta	4	
QM	26,826	**TUBB**	27,900	4.7	1.37	tubulin beta, partial	4; 7	
QM	1360	**ACTB**	14,615	5.0	1.21	actin, cytoplasmic, partial	4; 6	
QM	821	**TPM1**	34,308	4.6	1.18	tropomyosin-1	1; 4	Ca
QM	869	CFAP161	33,433	6.0	0.73	cilia- and flagella-associated protein 161	4	
QM	1349	**CALM1**	17,827	4.1	0.73	calmodulin	4; 7	Ca
QM	946	**TPM4**	30,326	4.7	0.70	tropomyosin-4	4	Ca
QM	435	**LMNA**	55,491	5.3	0.61	60 kDa neurofilament protein-like	1; 4; 5; 6; 7	Ca
QM	14,602	NUDC	44,488	5.1	0.61	nuclear migration protein nudC	4	
QM	638	**ACTG1**	41,797	5.2	0.60	actin beta/gamma 1	4	
QM	1331	**TUBB**	19,149	4.7	0.58	tubulin beta, first part	4; 7	
QM	624	**ACTG1**	42,449	4.6	0.56	actin beta/gamma 1	4	
QM	431	**LMNA**	55,663	5.3	0.52	60 kDa neurofilament protein-like	1; 4; 5; 6; 7	Ca
QM	820	ANXA13	34,910	4.7	0.51	annexin A13		Ca
QM	570	ILK	46,517	6.4	0.51	integrin-linked protein kinase	4	
QM	815	**TUBB**	34,832	5.1	0.51	tubulin beta, partial	4; 7	
QM	2254	**CCDC151**	56,609	5.7	0.49	coiled-coil domain-containing protein 151	4	
QM	663	**TUBA1A**	41,535	5.7	0.48	tubulin alpha-1A chain	4	
QM	1256	**DPYSL2**	27,335	6.3	0.44	dihydropyrimidinase-like, partial	4	
QM	1011	**ACTB**	29,008	5.4	0.42	actin, cytoplasmic, partial	4; 6	
QM	133	CLCA4	93,882	5.8	0.36	calcium-activated chloride channel regulator 4A		Ca
**Part C. Redox and detoxification (GO:0006790_ Sulphur compound metabolic process in bold)**								
Sp.	label	abbrev.	Mr	pI	Var.	Identification	G.	Ca
ZM	4237	TALDO1	38,290	4.8	2.61	transaldolase		
ZM	4204	**GLRX3**	39,207	4.8	2.47	glutaredoxin-3	2	
ZM	686	**CTH**	42,496	6.0	1.54	cystathionine gamma-lyase	1; 2; 3; 6	Ca
ZM	81,959	**PAPSS1**	67,505	6.6	1.50	bifunctional 3′-phosphoadenosine 5′-phosphosulfate synthase	2	
ZM	644	ALDH1A2	44,166	5.7	1.47	retinal dehydrogenase 2	3; 6	
ZM	830	**SULT1A1**	37,942	5.5	1.32	sulfotransferase family 1A member 1	2	
ZM	81,979	**PAPSS1**	65,696	6.7	1.28	bifunctional 3′-phosphoadenosine 5′-phosphosulfate synthase	2	
ZM	650	ADH5	44,380	4.67	1.25	NADP-dependent alcohol dehydrogenase C	3	
ZM	639	**BPNT1**	44,175	5.8	1.23	3′(2′),5′-bisphosphate nucleotidase 1	2	
ZM	1176	**SOD2**	24,887	8.5	0.86	superoxide dismutase [Mn], mitochondrial	1; 2; 6	
ZM	1105	**GSTZ1**	26,883	5.7	0.75	maleylacetoacetate isomerase	2; 3	
ZM	517	CNDP2	48,800	5.5	0.68	cytosolic non-specific dipeptidase		
QM	1127	CNDP2	27,369	4.7	1.66	cytosolic non-specific dipeptidase		
QM	518	ALDH1A2	48,664	6.1	1.59	retinal dehydrogenase 2	3; 6	
QM	2530	**SULT1A1**	33,864	5.9	1.48	sulfotransferase family 1A member 1	2	
QM	1099	**GST**	27,684	5.9	1.38	glutathione S-transferase-like	1; 2; 3	
QM	669	**CTH**	41,119	6.1	1.37	cystathionine gamma-lyase	1; 2; 3; 6	Ca
QM	694	**CTH**	40,865	6.2	1.35	cystathionine gamma-lyase	1; 2; 3; 6	Ca
QM	1061	**GSTZ1**	27,873	5.4	1.23	maleylacetoacetate isomerase	1; 2; 3	
QM	1303	**SOD1**	21,470	5.9	0.87	superoxide dismutase [Cu/Zn]	1; 2; 4; 6; 7	
QM	558	**GSS**	45,618	5.8	0.77	glutathione synthetase	2; 3	
QM	1353	CYB5A	18,002	4.7	0.73	cytochrome b5	3	
QM	1351	NENF	17,075	4.7	0.71	neudesin		
QM	1028	PRDX2	28,889	6.3	0.70	peroxiredoxin-like	1; 6	
QM	840	**GSR**	34,032	5.9	0.65	glutathionyl-hydroquinone reductase YqjG	1; 2	
QM	1321	**AHCY**	20,643	6.3	0.64	adenosylhomocysteinase B	2; 3	
QM	1184	PRXL2C	27,023	5.7	0.63	dye-decolourizing peroxidase YfeX	1	
QM	736	**GSTP1**	38,905	5.8	0.48	glutathione S-transferase P 1	1; 2; 3; 6; 7	
QM	578	ADSS2	46,200	6.3	0.47	adenylosuccinate synthetase	3	
**Part D. Energy and metabolism (GO:0019752_ Carboxylic acid metabolic process in bold)**								
Sp.	label	abbrev.	Mr	pI	Var.	Identification	G.	Ca
ZM	12,452	NTPCR	26,212	5.7	2.46	cancer-related nucleoside-triphosphatase		
ZM	1159	GPD2	26,110	6.6	2.01	glycerol-3-phosphate dehydrogenase, mitochondrial		Ca
ZM	71,084	**ATIC**	66,279	5.9	1.65	bifunctional purine biosynthesis protein PURH	3	
ZM	242	**PCCA**	76,341	6.7	1.49	propionyl-CoA carboxylase alpha chain, mitochondrial	3	
ZM	20,205	**FAH**	44,598	5.6	1.46	fumarylacetoacetase	3	
ZM	731	**PCK1_2**	41,321	5.9	1.46	phosphoenolpyruvate carboxykinase [GTP], partial	1; 3	
ZM	607	**FH**	45,269	6.0	1.45	fumarate hydratase, mitochondrial	1; 3	
ZM	14,678	ALDOB	28,111	5.7	1.42	fructose-1, 6-bisphosphate aldolase	3	
ZM	114	**ALDH1L1**	92,374	6.0	1.41	cytosolic 10-formyltetrahydrofolate dehydrogenase	3	
ZM	1670	SPATA18	70,773	5.5	1.41	mitochondria-eating protein	1	
ZM	1339	ATP5F1D	12,048	4.4	1.23	ATP synthase delta chain, mitochondrial		
ZM	729	**HPD**	41,675	5.7	1.23	4-hydroxyphenylpyruvate dioxygenase	3	
ZM	720	MYG1	41,996	5.5	1.21	UPF0160 protein MYG1, mitochondrial		
ZM	812	**HIBCH**	38,744	6.2	0.78	3-hydroxyisobutyryl-CoA hydrolase, mitochondrial	3	
ZM	641	**CKB**	43,718	7.0	0.71	arginine kinase	3	
ZM	532	PCYT2	47,622	6.3	0.70	ethanolamine-phosphate cytidylyltransferase		
ZM	318	**PCK1**	71,984	6.7	0.66	phosphoenolpyruvate carboxykinase, cytosolic [GTP]	1; 3	
ZM	20,062	**LDHB**	42,852	5.6	0.64	opine/octopine dehydrogenase, tauropine dehydrogenase	3	
ZM	272	**GBA2**	79,092	4.6	0.62	glucosidase 2 subunit beta	3	
QM	609	TKTL2_short	44,391	6.1	2.67	transketolase protein 2, partial		
QM	2367	**CS**	40,656	6.4	2.47	citrate synthase, mitochondrial	3	
QM	276	**PCK1**	69,690	6.4	2.25	phosphoenolpyruvate carboxykinase [GTP]	1; 3	
QM	611	AK5	43,717	5.8	2.24	adenylate kinase isoenzyme 5		
QM	811	**MDH1B**	35,684	6.3	2.06	malate dehydrogenase, cytosolic	3	
QM	1217	**MDH1B**	27,069	4.7	1.96	malate dehydrogenase, cytosolic	3	
QM	792	**GAPDH**	35,711	6.2	1.84	glyceraldehyde-3-phosphate dehydrogenase	3; 4; 5	
QM	420	**MCCC2**	56,870	6.7	1.76	3-methylcrotonoyl-CoA carboxylase beta chain, mitochondrial	2; 3	
QM	752	**PCK1_2**	37,948	5.8	1.69	phosphoenolpyruvate carboxykinase [GTP]	1; 3	
QM	1385	NIPSNAP1	14,360	6.5	1.49	protein NipSnap	7	Ca
QM	513	**SHMT1**	49,003	6.5	1.47	serine hydroxymethyltransferase, cytosolic	3; 5	
QM	855	IMPA1	33,752	5.4	1.40	inositol monophosphatase		
QM	1358	COX6B1	16,778	6.0	0.78	cytochrome c oxidase subunit 6B1		
QM	932	PHB1	31,386	5.4	0.76	prohibitin	6	
QM	517	**ENO1**	48,104	5.8	0.76	enolase	3; 6	
QM	616	SPATA18	43,782	5.3	0.69	mitochondria-eating protein	1	
QM	1408	ATP5F1D	12,863	4.6	0.65	ATP synthase delta chain, mitochondrial		
QM	828	**ENO1_short**	34,988	5.5	0.64	enolase-short form alias c-myc promoter-binding protein-1	3; 6	
QM	1281	**MDH2**	24,327	6.4	0.64	malate dehydrogenase, mitochondrial	3	
QM	501	ATP5F1B	50,871	5.1	0.61	ATP synthase subunit beta, mitochondrial		
QM	7190	**ENO1**	39,080	5.5	0.58	enolase	3; 6	
QM	314	TKTL2	65,910	6.0	0.56	transketolase protein 2		
QM	655	**PCCB**	41,805	5.3	0.45	propionyl-CoA carboxylase beta chain, mitochondrial	2; 3	
**Part E. Transcription/translation (GO:0006417_ Regulation of translation in bold)**								
Sp.	label	abbrev.	Mr	pI	Var.	Identification	G.	Ca
ZM	995	**EIF3F**	29,818	5.4	1.71	eukaryotic translation initiation factor 3 subunit F	5	
ZM	352	**DDX3X**	66,641	6.1	1.69	ATP-dependent RNA helicase DDX3X	1; 5; 6; 7	
ZM	819	**EEF2_short**	38,350	5.9	1.58	elongation factor 2, partial	1; 5; 7	
ZM	19,977	FAM172A	44,808	5.8	1.54	cotranscriptional regulator FAM172A		
ZM	474	U2AF2	53,138	5.6	1.48	splicing factor U2AF 50 kDa subunit		
ZM	356	FARSB	69,076	5.4	1.46	phenylalanine-tRNA ligase beta subunit	3	
ZM	629	**PA2G4**	44,025	6.3	1.41	proliferation-associated protein 2G4	5; 6; 7	
ZM	260	FUBP1	76,901	6.0	1.27	far upstream element-binding protein 1		
ZM	1259	**EIF5A**	20,524	5.0	1.18	eukaryotic translation initiation factor 5A	5; 6	
ZM	649	WARS1	43,779	5.96	0.70	tryptophanyl-tRNA synthetase, cytoplasmic	3	
ZM	431	NAP1L1	60,086	4.3	0.68	nucleosome assembly protein 1 1		
ZM	81	**EEF2**	93,962	7.5	0.65	elongation factor 2	1; 5	
QM	644	WARS1	42,768	5.8	2.44	tryptophanyl-tRNA synthetase, cytoplasmic	3	
QM	710	**EIF4A1**	39,889	5.8	1.60	eukaryotic initiation factor 4A-I	5	
QM	991	**EIF4B**	29,725	5.6	1.44	eukaryotic translation initiation factor 4B	5	
QM	614	**PA2G4**	43,956	6.3	1.39	proliferation-associated protein 2G4	3; 5; 7	
QM	952	**EIF3F**	30,865	5.5	1.28	eukaryotic translation initiation factor 3 subunit F	5	
QM	261	FUBP1	70,697	5.7	0.68	far upstream element-binding protein 1		
QM	780	**DDX3X**	37,130	6.3	0.66	ATP-dependent RNA helicase DDX3X	1; 5; 6; 7	
QM	1559	ELOB	21,107	4.6	0.65	elongin B	1	
QM	1422	**RIDA**	11,327	6.0	0.40	2-iminobutanoate/2-iminopropanoate deaminase	3; 5	

**Legend:** Sp., ZM for zebra mussel, QM for quagga mussel; labels correspond to the reference number of the DAP in the proteomic analysis; abbrev. refer to GeneCards abbreviation by similarity; Mr and pI are observed DAP molecular mass and isoelectric point, respectively; var. greater than 1 indicates an increase in abundance of proteoforms after cadmium exposure, and conversely, a decrease for values lower than 1. Table 1 presents the five functional categories: chaperone, processing, folding, and degradation (A); calcium and cytoskeleton alterations (B); redox and detoxification (C); energy and metabolism (D); transcription and translation (E). G. columns refer to Gene Ontology terms: G1. GO:0033554_Cellular response to stress; G2. GO:0006790_Sulfur compound metabolic process; G3. GO:0019752_Carboxylic acid metabolic process; G4. GO:0005856_Cytoskeleton and G5. GO:0006417_Regulation of translation; G6. GO:0042981_Regulation of apoptotic process; G7. GO:0045055_Regulated exocytosis; Ca for calcium dependant protein. A colour version including observed MW and pI is proposed in Table S3.

**Table 2:** DAPs sharing similar protein identities.

Variations		Abbreviation	Protein
Abundances change in the same direction	Up	ALDH1A2	retinal dehydrogenase 2
		CTH	cystathionine gamma-lyase
		EIF3F	eukaryotic translation initiation factor 3 subunit F
		FLNA	filamin-A, partial
		HSPD1	heat shock protein 60, mitochondrial
		PA2G4	proliferation-associated protein 2G4
		PCK1	phosphoenolpyruvate carboxykinase [GTP], partial
		SULT1A1	sulfotransferase family 1A member 1
	Down	LMNA	60 kDa neurofilament protein-like
		MVP	major vault protein
		P4HB	protein disulfide-isomerase
		TUBA	tubulin alpha subunit
Abundances change in opposite directions	ZM Up QM Down	ATP5F1D	ATP synthase delta chain, mitochondrial
		DDX3X	ATP-dependent RNA helicase DDX3X
		FUBP1	far upstream element-binding protein 1
		SPATA18	mitochondria-eating protein
	ZM Down QM Up	CNDP2	cytosolic non-specific dipeptidase
		CNN3	calponin homolog, protein unc-87
		GSTZ1	maleylacetoacetate isomerase alias GST Z
		WARS1	tryptophanyl-tRNA synthetase, cytoplasmic
		PCK1	phosphoenolpyruvate carboxykinase, cytosolic [GTP]
		TUBB	tubulin beta, partial

## Data Availability

The data are public on the figshare.com website: https://figshare.com/projects/Cadmium_highlights_common_and_specific_responses_of_the_two_dressenid_species_Dreissena_polymorpha_and_Dreissena_rostri-formis_bugensis/198700 (accessed on 18 March 2024).

## Supplementary Materials

The following are available online at https://www.mdpi.com/article/10.3390/proteomes12020010/s1, Table S1: MS/MS identification of zebra mussel differentially abundant proteoforms after cadmium exposure. Table S2: MS/MS identification of quagga mussel differentially abundant proteoforms after cadmium exposure. Table S3: Colour version of Table 1 including observed MW and pI. Table S4: Gene Ontology Biological Process enrichment in zebra and quagga mussel gills after cadmium exposure. Table S5: Short Summary of protein functions extracts from online database annotations [19,20,21,22,23,24,25,26,27,28,29,30,31,32,33,34,35,36,37,38,39,40,41,42,43,44,45,46,47,48,49,50,51,52,53,54,55,56,57,58,59,60,61,62,63,64,65,66,67,68,69,70,71,72,73,74,75,76,77,78,79,80,81,82,83,84,85,86,87,88,89,90,91,92,93,94,95,96,97,98,99,100,101,102,103,104,105,106,107,108,109,110,111,112]. Figure S1: Water physicochemical parameters during the 7-day of cadmium exposure. Figure S2: images of selected 2DE gels of Zebra Mussel gill proteome. Figure S3: images of selected 2DE gels of Quagga Mussel gill proteome. Figure S4: Distribution of ZM_DAPs based on main GO terms in zebra mussel (ZM) gills after cadmium exposure. Figure S5: Distribution of QM_DAPs based on main GO terms in quagga mussel (QM) gills after cadmium exposure. Figure S6: Evidence of cytoskeleton short proteoforms revealed by cadmium exposure. Figure S7: Comparison of HSP70 proteoforms in quagga mussel (QM).
