# Supplementary material for: Cadmium Highlights Common and Specific Responses of Two Freshwater Sentinel Species, Dreissena polymorpha and Dreissena rostriformis bugensis
Source: Proteomes. 2024 Mar 26;12(2):10. doi: 10.3390/proteomes12020010 (PMC11036304; doi:10.3390/proteomes12020010)
Supplement: Supplementary file 1 [file proteomes-12-00010-s001.zip › FBultelle Supplementary files 20Feb24/FBultelle Supplementary Figures-20Feb24.pptx]

## Slide 1
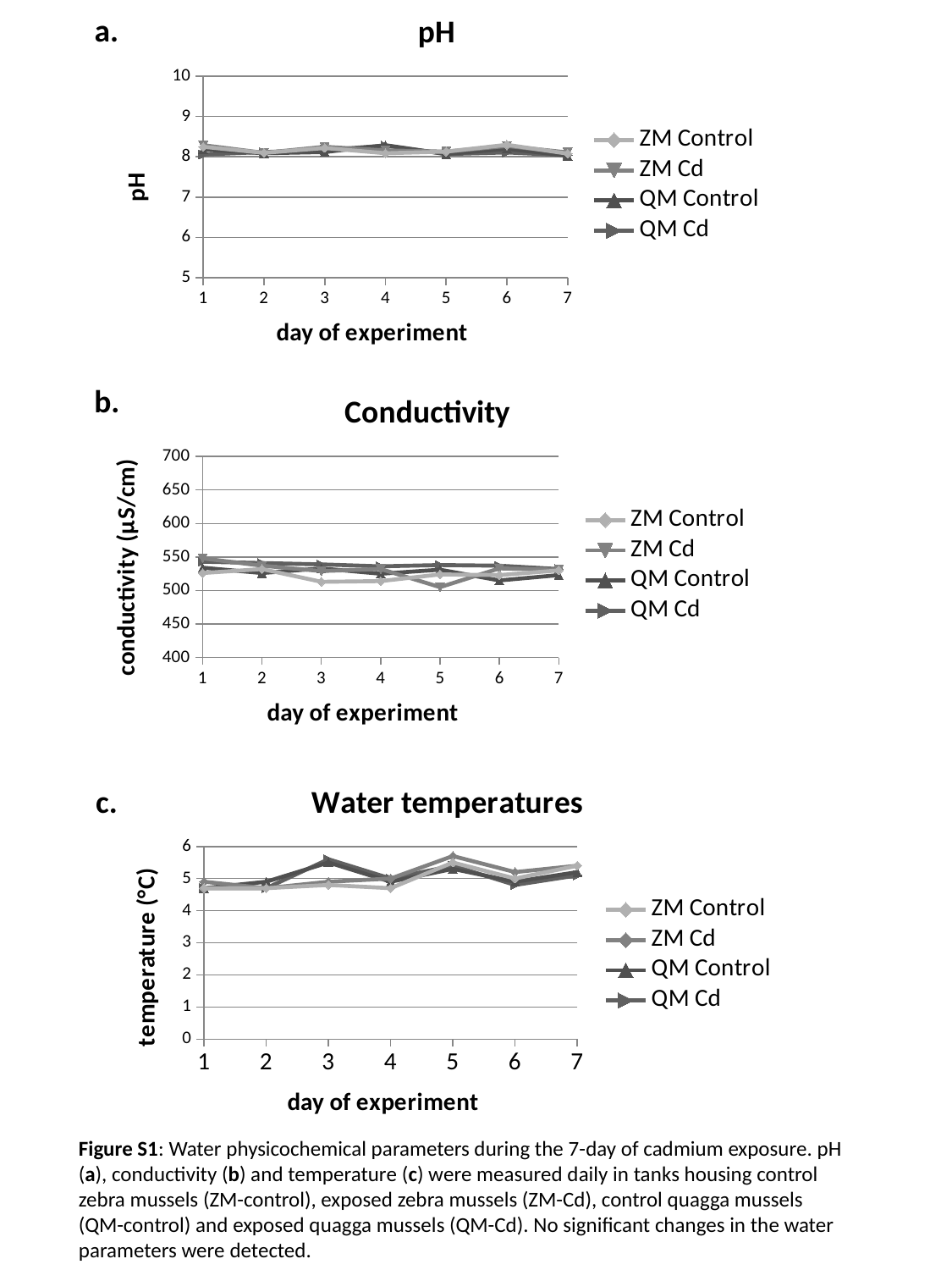

### Chart: pH
| Category | QM Cd | ZM Control | ZM Cd | QM Control |
|---|---|---|---|---| a.
 b.
### Chart: Conductivity
| Category | QM Cd | ZM Control | ZM Cd | QM Control |
|---|---|---|---|---|
### Chart: Water temperatures
| Category | QM Cd | ZM Control | ZM Cd | QM Control |
|---|---|---|---|---| c.
Figure S1: Water physicochemical parameters during the 7-day of cadmium exposure. pH (a), conductivity (b) and temperature (c) were measured daily in tanks housing control zebra mussels (ZM-control), exposed zebra mussels (ZM-Cd), control quagga mussels (QM-control) and exposed quagga mussels (QM-Cd). No significant changes in the water parameters were detected.

## Slide 2
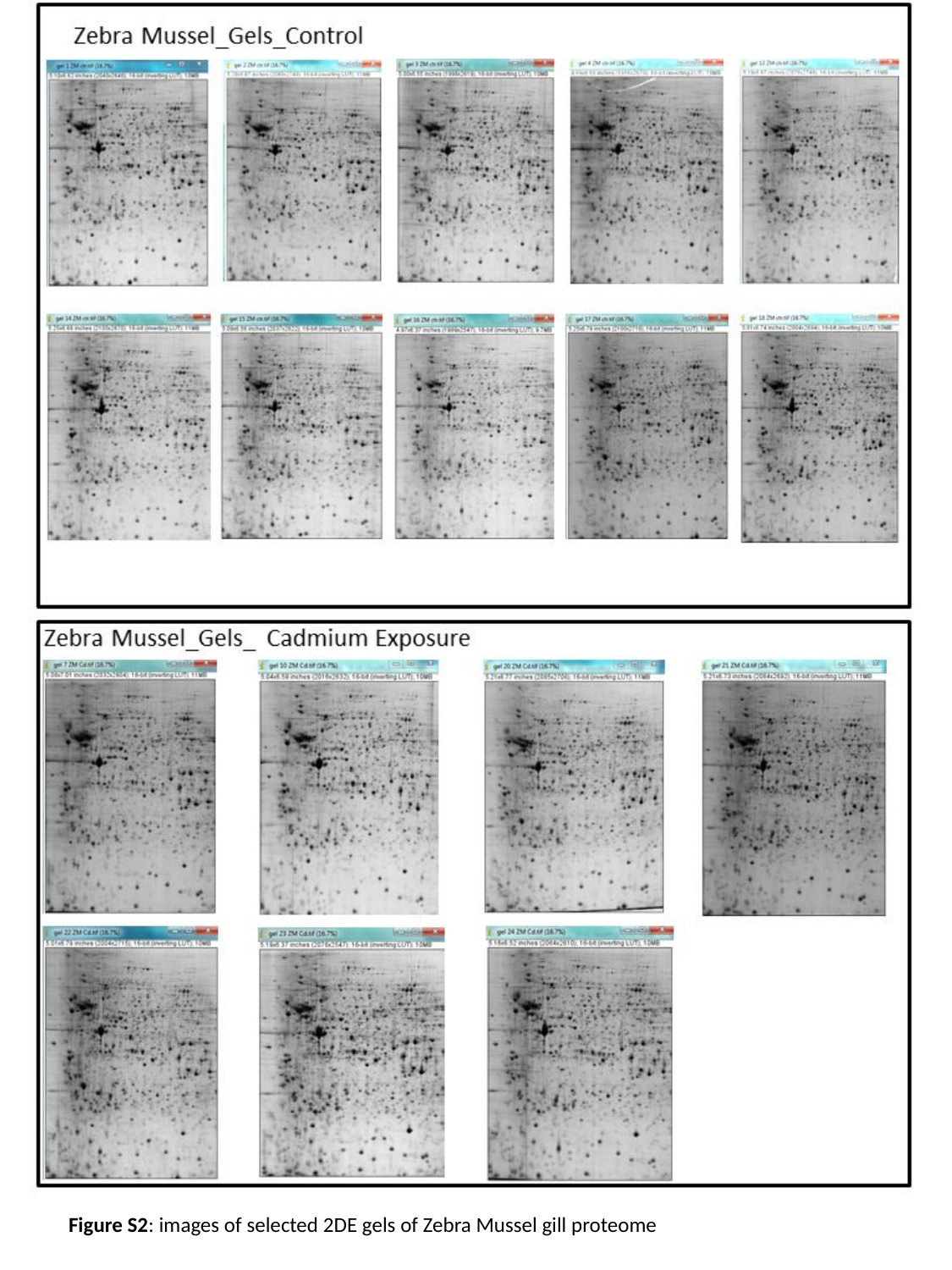

Figure S2: images of selected 2DE gels of Zebra Mussel gill proteome

## Slide 3
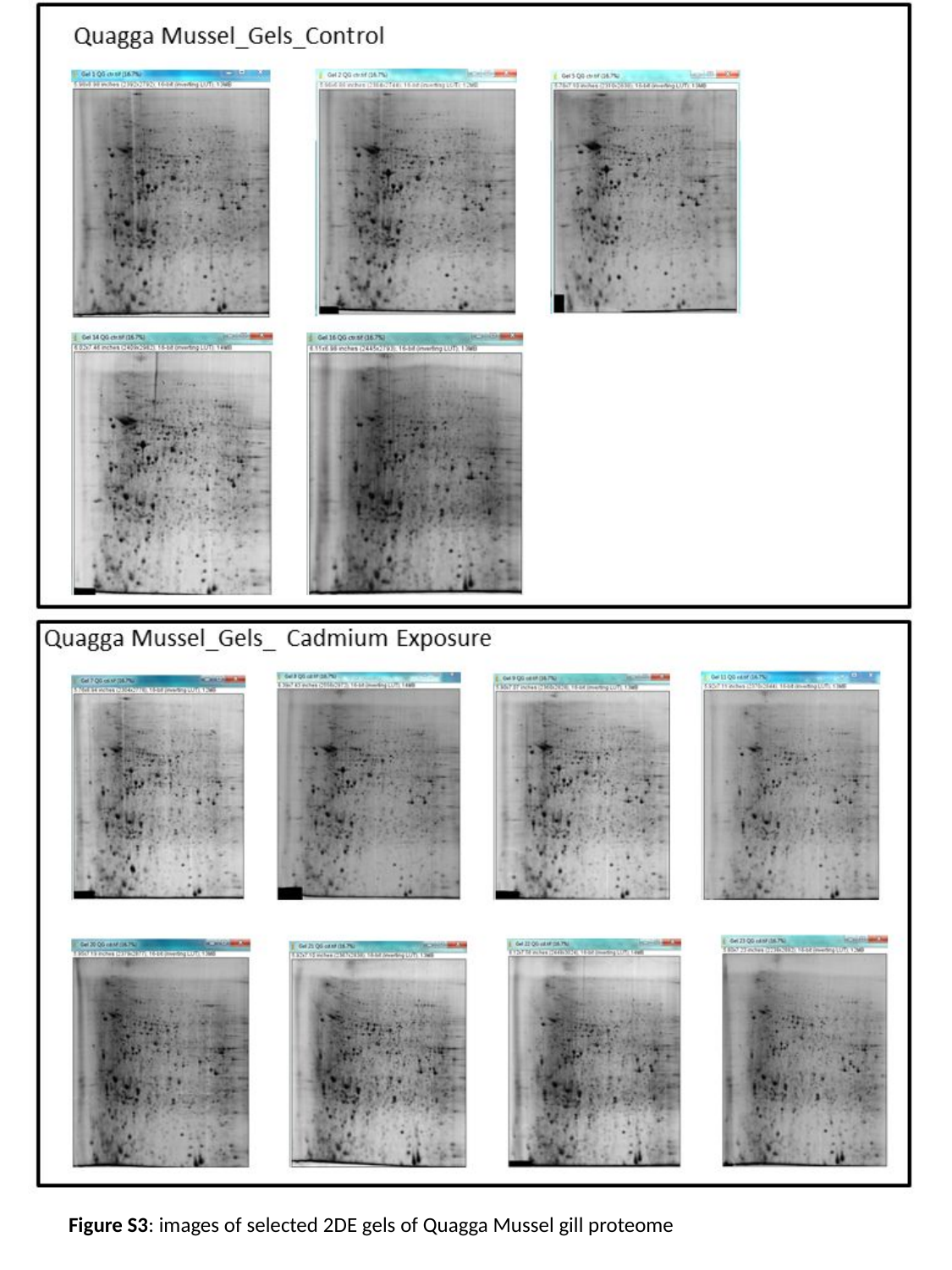

Figure S3: images of selected 2DE gels of Quagga Mussel gill proteome

## Slide 4
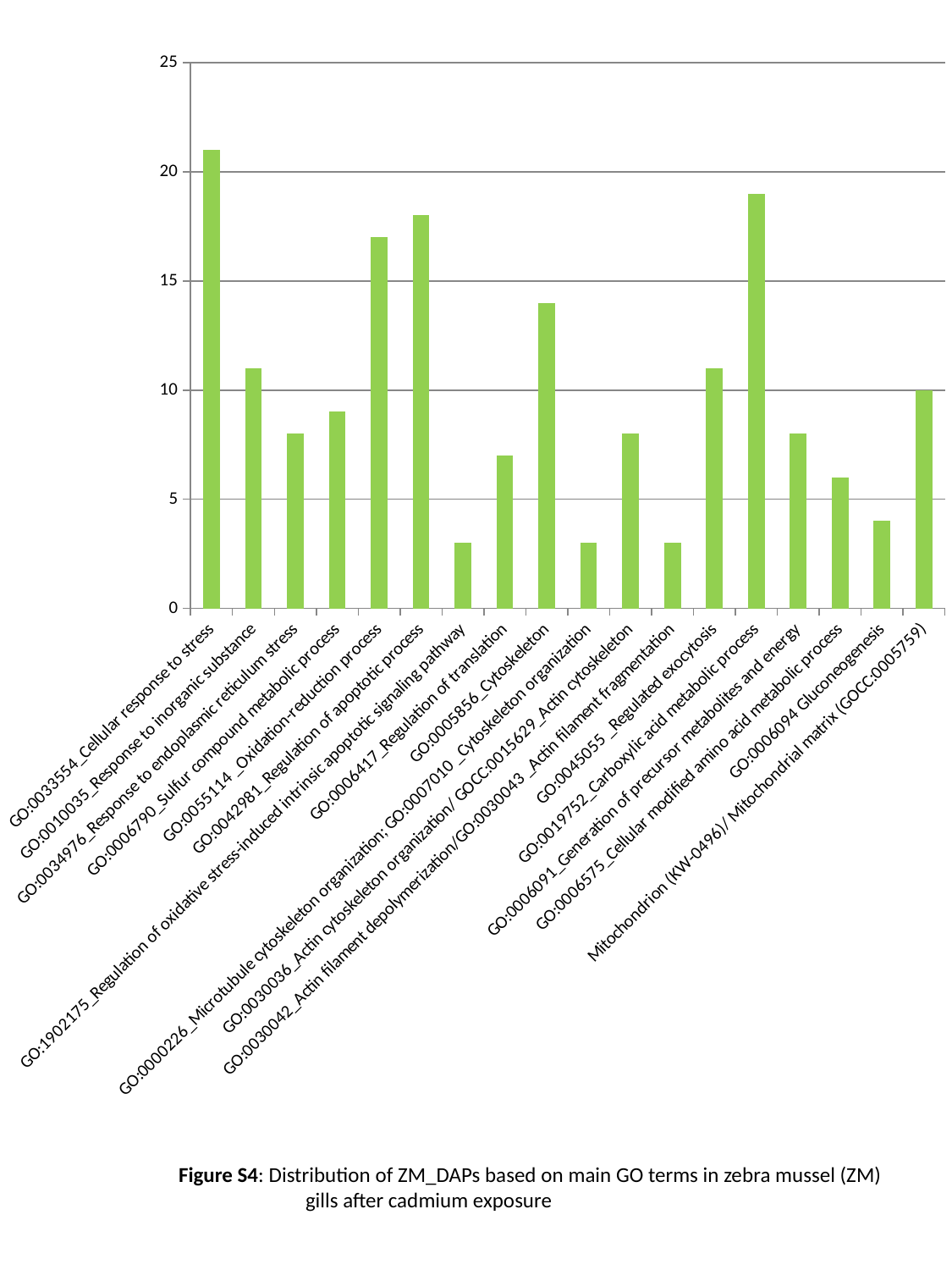

### Chart
| Category | ZM_DAPs |
|---|---|
| GO:0033554_Cellular response to stress | 21.0 |
| GO:0010035_Response to inorganic substance | 11.0 |
| GO:0034976_Response to endoplasmic reticulum stress | 8.0 |
| GO:0006790_Sulfur compound metabolic process | 9.0 |
| GO:0055114 _Oxidation-reduction process | 17.0 |
| GO:0042981_Regulation of apoptotic process | 18.0 |
| GO:1902175_Regulation of oxidative stress-induced intrinsic apoptotic signaling pathway | 3.0 |
| GO:0006417_Regulation of translation | 7.0 |
| GO:0005856_Cytoskeleton | 14.0 |
| GO:0000226_Microtubule cytoskeleton organization; GO:0007010 _Cytoskeleton organization | 3.0 |
| GO:0030036_Actin cytoskeleton organization/ GOCC:0015629_Actin cytoskeleton | 8.0 |
| GO:0030042_Actin filament depolymerization/GO:0030043 _Actin filament fragmentation | 3.0 |
| GO:0045055 _Regulated exocytosis | 11.0 |
| GO:0019752_Carboxylic acid metabolic process | 19.0 |
| GO:0006091_Generation of precursor metabolites and energy | 8.0 |
| GO:0006575_Cellular modified amino acid metabolic process | 6.0 |
| GO:0006094 Gluconeogenesis | 4.0 |
| Mitochondrion (KW-0496)/ Mitochondrial matrix (GOCC:0005759) | 10.0 |Figure S4: Distribution of ZM_DAPs based on main GO terms in zebra mussel (ZM) 	gills after cadmium exposure

## Slide 5
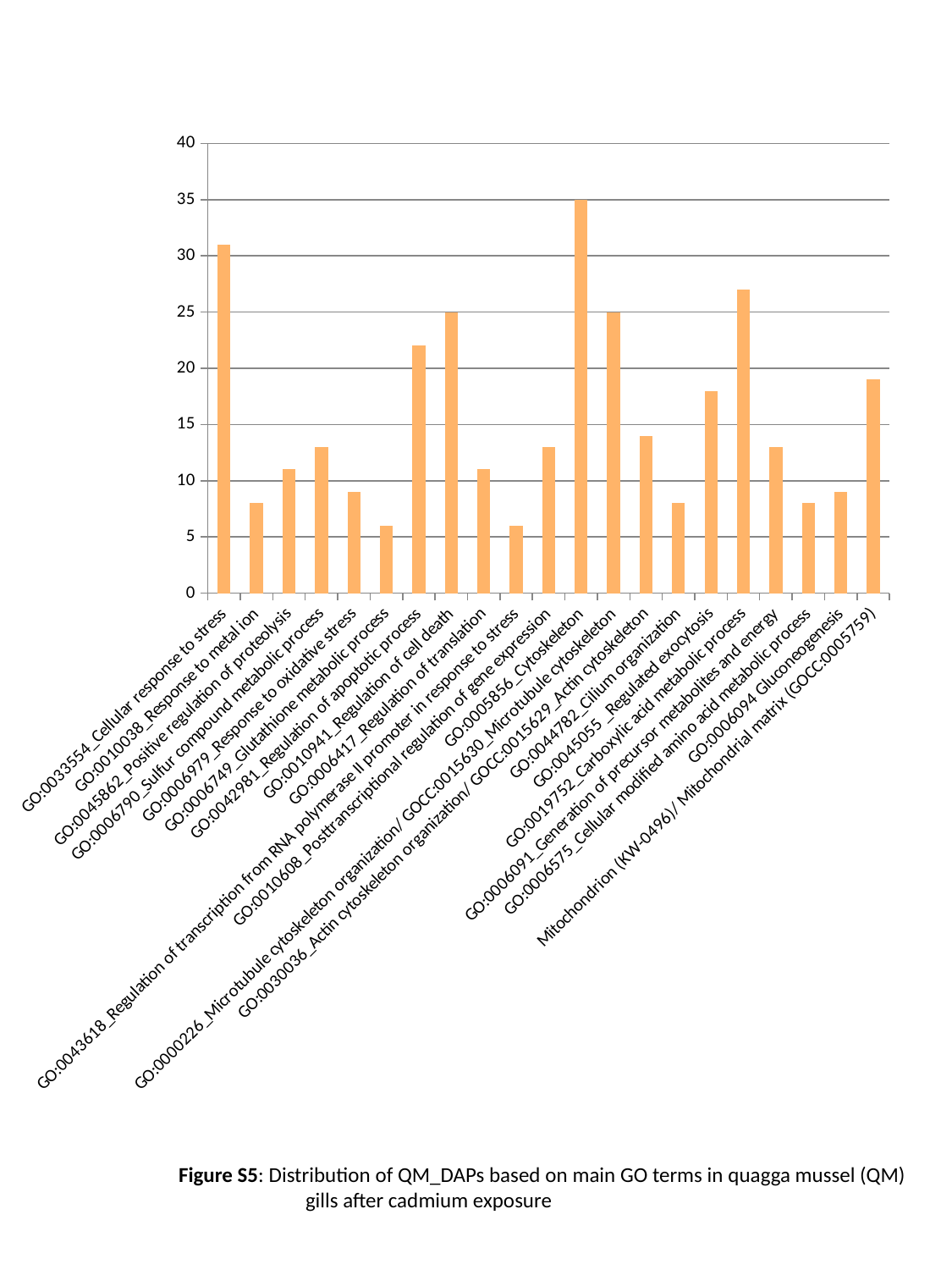

### Chart
| Category | ZM_DAPs |
|---|---|
| GO:0033554_Cellular response to stress | 31.0 |
| GO:0010038_Response to metal ion | 8.0 |
| GO:0045862_Positive regulation of proteolysis | 11.0 |
| GO:0006790_Sulfur compound metabolic process | 13.0 |
| GO:0006979_Response to oxidative stress | 9.0 |
| GO:0006749_Glutathione metabolic process | 6.0 |
| GO:0042981_Regulation of apoptotic process | 22.0 |
| GO:0010941_Regulation of cell death | 25.0 |
| GO:0006417_Regulation of translation | 11.0 |
| GO:0043618_Regulation of transcription from RNA polymerase II promoter in response to stress | 6.0 |
| GO:0010608_Posttranscriptional regulation of gene expression | 13.0 |
| GO:0005856_Cytoskeleton | 35.0 |
| GO:0000226_Microtubule cytoskeleton organization/ GOCC:0015630_Microtubule cytoskeleton | 25.0 |
| GO:0030036_Actin cytoskeleton organization/ GOCC:0015629_Actin cytoskeleton | 14.0 |
| GO:0044782_Cilium organization | 8.0 |
| GO:0045055 _Regulated exocytosis | 18.0 |
| GO:0019752_Carboxylic acid metabolic process | 27.0 |
| GO:0006091_Generation of precursor metabolites and energy | 13.0 |
| GO:0006575_Cellular modified amino acid metabolic process | 8.0 |
| GO:0006094 Gluconeogenesis | 9.0 |
| Mitochondrion (KW-0496)/ Mitochondrial matrix (GOCC:0005759) | 19.0 |Figure S5: Distribution of QM_DAPs based on main GO terms in quagga mussel (QM) 	gills after cadmium exposure

## Slide 6
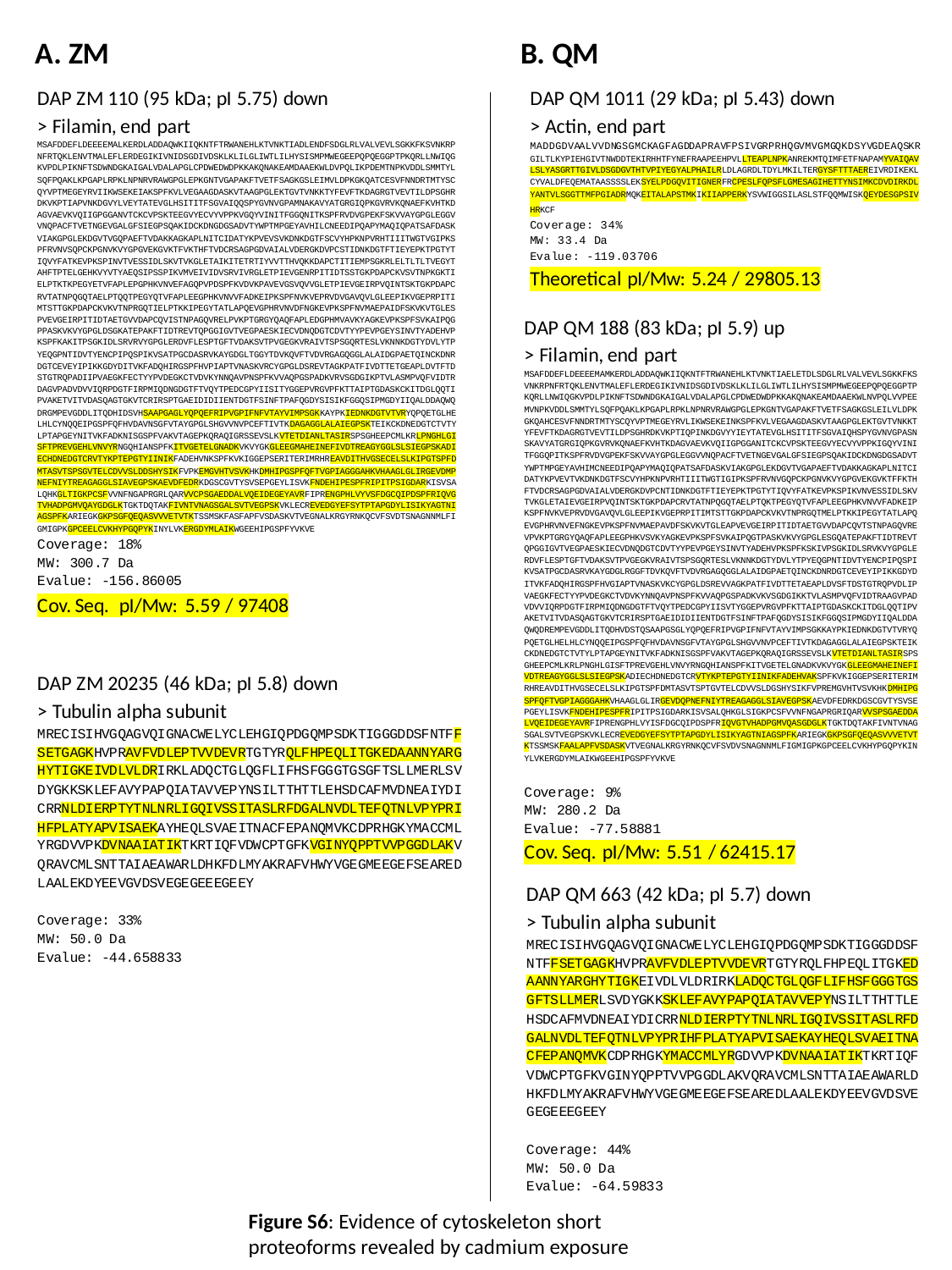

Figure S6: Evidence of cytoskeleton short proteoforms revealed by cadmium exposure

## Slide 7
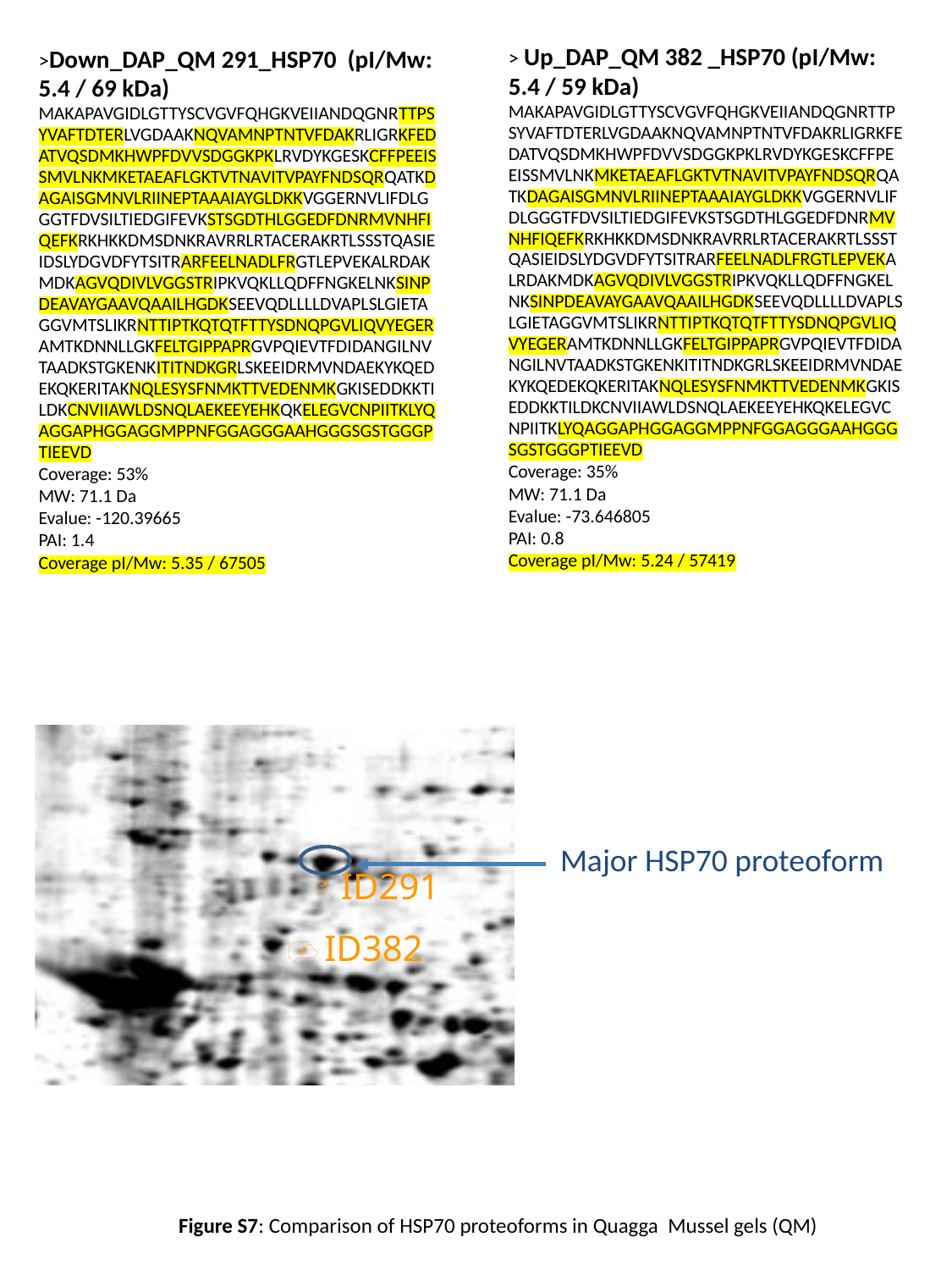

> Up_DAP_QM 382 _HSP70 (pI/Mw: 5.4 / 59 kDa)
MAKAPAVGIDLGTTYSCVGVFQHGKVEIIANDQGNRTTPSYVAFTDTERLVGDAAKNQVAMNPTNTVFDAKRLIGRKFEDATVQSDMKHWPFDVVSDGGKPKLRVDYKGESKCFFPEEISSMVLNKMKETAEAFLGKTVTNAVITVPAYFNDSQRQATKDAGAISGMNVLRIINEPTAAAIAYGLDKKVGGERNVLIFDLGGGTFDVSILTIEDGIFEVKSTSGDTHLGGEDFDNRMVNHFIQEFKRKHKKDMSDNKRAVRRLRTACERAKRTLSSSTQASIEIDSLYDGVDFYTSITRARFEELNADLFRGTLEPVEKALRDAKMDKAGVQDIVLVGGSTRIPKVQKLLQDFFNGKELNKSINPDEAVAYGAAVQAAILHGDKSEEVQDLLLLDVAPLSLGIETAGGVMTSLIKRNTTIPTKQTQTFTTYSDNQPGVLIQVYEGERAMTKDNNLLGKFELTGIPPAPRGVPQIEVTFDIDANGILNVTAADKSTGKENKITITNDKGRLSKEEIDRMVNDAEKYKQEDEKQKERITAKNQLESYSFNMKTTVEDENMKGKISEDDKKTILDKCNVIIAWLDSNQLAEKEEYEHKQKELEGVCNPIITKLYQAGGAPHGGAGGMPPNFGGAGGGAAHGGGSGSTGGGPTIEEVD
Coverage: 35%
MW: 71.1 Da
Evalue: -73.646805
PAI: 0.8
Coverage pI/Mw: 5.24 / 57419
>Down_DAP_QM 291_HSP70 (pI/Mw: 5.4 / 69 kDa)
MAKAPAVGIDLGTTYSCVGVFQHGKVEIIANDQGNRTTPSYVAFTDTERLVGDAAKNQVAMNPTNTVFDAKRLIGRKFEDATVQSDMKHWPFDVVSDGGKPKLRVDYKGESKCFFPEEISSMVLNKMKETAEAFLGKTVTNAVITVPAYFNDSQRQATKDAGAISGMNVLRIINEPTAAAIAYGLDKKVGGERNVLIFDLGGGTFDVSILTIEDGIFEVKSTSGDTHLGGEDFDNRMVNHFIQEFKRKHKKDMSDNKRAVRRLRTACERAKRTLSSSTQASIEIDSLYDGVDFYTSITRARFEELNADLFRGTLEPVEKALRDAKMDKAGVQDIVLVGGSTRIPKVQKLLQDFFNGKELNKSINPDEAVAYGAAVQAAILHGDKSEEVQDLLLLDVAPLSLGIETAGGVMTSLIKRNTTIPTKQTQTFTTYSDNQPGVLIQVYEGERAMTKDNNLLGKFELTGIPPAPRGVPQIEVTFDIDANGILNVTAADKSTGKENKITITNDKGRLSKEEIDRMVNDAEKYKQEDEKQKERITAKNQLESYSFNMKTTVEDENMKGKISEDDKKTILDKCNVIIAWLDSNQLAEKEEYEHKQKELEGVCNPIITKLYQAGGAPHGGAGGMPPNFGGAGGGAAHGGGSGSTGGGPTIEEVD
Coverage: 53%
MW: 71.1 Da
Evalue: -120.39665
PAI: 1.4
Coverage pI/Mw: 5.35 / 67505
ID291
ID382
Major HSP70 proteoform
Figure S7: Comparison of HSP70 proteoforms in Quagga Mussel gels (QM)
